# A circuit that integrates drive state and social contact to gate mating

**DOI:** 10.1038/s41586-025-09327-x

**Published:** 2025-09-03

**Authors:** Lindsey D. Salay, Doris Y. Tsao, David J. Anderson

**Affiliations:** 1 Division of Biology and Biological Engineering, California Institute of Technology, Pasadena, CA USA; 2 Tianqiao and Chrissy Chen Institute for Neuroscience Caltech, Pasadena, CA USA; 3 Howard Hughes Medical Institute, Chevy Chase, MD USA; 4 Department of Molecular and Cell Biology, UC Berkeley, Berkeley, CA USA

**Keywords:** Sexual behaviour, Sensory processing

## Abstract

Internal motive states, such as sexual arousal, drive behaviour in response to social cues. However, little is known about how internal states and external cues are integrated to release appropriate behaviours at the correct moment during a social interaction, such as the transition from the appetitive to the consummatory phases of mating^1,2^. Here we identify a neural circuit in male mice that gates the onset of consummatory reproductive behaviours on contact with a mating partner. Stimulating MPOA^*Esr1*∩*Vgat*^ hypothalamic neurons promotes mounting of conspecifics and three-dimensional dummy objects^3^. We find that such mounting depends on mechanosensory but not visual cues. Through a large-scale electrophysiological screen, we identify neurons in the subparafascicular thalamic nucleus that nonlinearly integrate medial preoptic area of the hypothalamus (MPOA) and mechanosensory input to encode contact with a potential mate. Circuit tracing and perturbations demonstrated that this conjunctive coding occurs by means of convergent disinhibition from MPOA and excitation from the spinal trigeminal nucleus. Functional manipulations and calcium recordings showed these social-contact neurons, marked by parathyroid hormone 2, were essential for and able to promote mounting. These data indicate that subparafascicular thalamic nucleus-parathyroid hormone 2 neurons integrate internal drive with social touch to trigger mounting at opportune moments during mating. More generally, our findings uncover a brain mechanism whereby an internal state can attribute a social quality to a generic touch to initiate purposeful reproductive actions.

## Abstract (web summary)

Analyses of consummatory reproductive behaviours in male mice uncover a brain mechanism whereby an internal state can attribute a social quality to a generic touch to initiate purposeful reproductive actions.

## Main

During a social interaction, animals need to decide not only how to act towards a conspecific, such as to fight a foe or mount a mate, but also when to release such consummatory behaviours in the course of an encounter^4–6^. This ability is critical for guiding successful social interactions and, thereby, reproductive success. Yet the mechanisms by which long-lasting internal drive states of ‘readiness’ are integrated with external cues to trigger behaviours at opportune moments remain unclear.

Such internal drive states can be artificially generated by stimulating hypothalamic neurons, including those that control aggression or mating^7–10^. For example, optogenetic stimulation of MPOA neurons evokes sexual arousal (indicated by singing)^11–15^, but only triggers consummatory actions when a suitable three-dimensional (3D) target is present^3,16,17^. Notably, stimulation can even evoke behaviours towards inanimate ‘attackable’ or ‘mountable’ objects^3,17–21^. Thus, whereas the nature of behaviours expressed is determined by the hypothalamic drive state, the release of consummatory behaviour is seemingly triggered by the target object itself, by means of features that indicate its identity, proximity or, in the case of social contexts, its actions^5,8^. These observations raise the questions of which object features govern the release of a given innate behavioural programme and how internal states shape this process.

Here we have identified a new neural circuit for male reproductive behaviours that gates the transition from the appetitive to the consummatory phase of mating^1,2^, initiating copulation. This circuit functions to encode moments of social contact in a sexual arousal-dependent manner to initiate mounting towards a desired partner. Our findings reveal how sensory cues and internal state are nonlinearly integrated by means of a disinhibitory gate, offering insights into the neural computations that time the release of goal-directed behaviours.

## Contact cues release hypothalamic drive

**Fig. 1:**
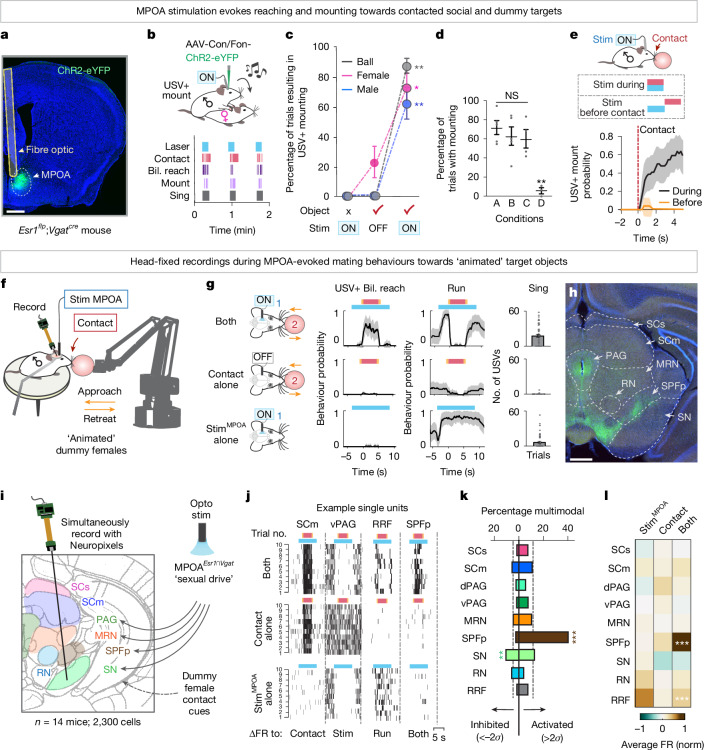
Multiregional recordings during MPOA-driven consummatory behaviours. **a**, Representative image of ChR2 expression in MPOA during MPOA^*Esr1*∩*Vgat*^ optogenetic stimulation. **b**, Schematic (top) and raster plots (bottom) of MPOA^*Esr1*∩*Vgat*^ optogenetic stimulation induced behaviours towards a female. Bil., bilateral; Con, Cre-ON; Fon, Flp-ON. **c**, Percentage of MPOA-stimulation (ON) or sham (OFF) trials resulting in mounting with USVs towards a female, male or inanimate object (*n* = 8 mice, 6 trials per mouse).Stim, stimulation. **d**, Percentage of MPOA-stimulation trials with mounting under white light (A), dark infrared light (B), whisker cuts/whiskerless (C) or face numb and whiskerless (D) conditions (*n* = 5 mice, 20 trials per mouse). **e**, Probability of mounting with MPOA stimulation delivered before (orange) or during object contact (grey; *n* = 5 mice, 10 trials per mouse). **f**, Schematic of the experimental model. **g**, Probability of USV+ bilateral reach (left) and run (middle) in MPOA stimulation alone, contact alone or both (stim^MPOA^ + contact) conditions (*n* = 5 mice, 10 trials per mouse). Number of USVs per trial (right). **h**, Representative image of MPOA^*Esr1*∩*Vgat*^ axons labelled with ChR2-eYFP. MRN, midbrain reticular nucleus; PAG, periaqueductal grey; RN, red nucleus; SN, substantia nigra. **i**, Schematic of probe trajectories. Opto, optogenetic. **j**, Spike rasters for four single units under the three conditions, ten trials per condition. FR, firing rate; RRF, retrorubral field; vPAG, ventral periaqueductal grey. **k**, Percentage of multimodal cells with significantly higher (right, more than 2*σ*) or lower activity (left, less than −2*σ*) under the multimodal condition relative to unimodal (*n* = 2,300 total single units from 14 mice; 10 trials per condition). Dashed line, mean of all cells. dPAG; dorsal periaqueductal grey. **l**, Mean population firing rate across each brain region during the three conditions. norm., normalized. *P < 0.05, **P < 0.01, ***P < 0.001. For all figure panels, data are mean ± standard error of the mean (s.e.m.). See Supplementary Table 2 for further details of the statistical analyses. Scale bars, 0.5 mm (**a**,**h**).

**Extended Data Fig. 1:**
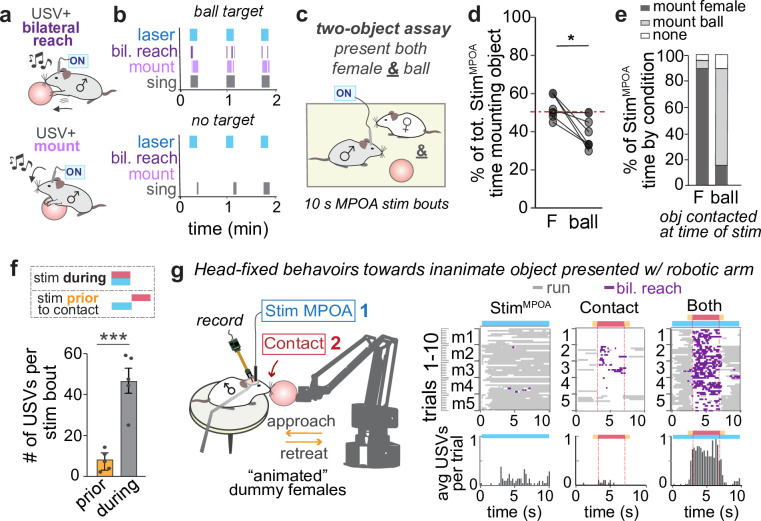
MPOA^*Esr1*∩*Vgat*^ stimulation invariantly induces mating behaviors towards contacted females and dummy females. **a-b**, Schematic (a) and raster plots (b) of MPOA-stimulation induced behaviors towards an inanimate object or in the absence of a target object. **c**, Schematic of behavioral paradigm involving access to both a female and a ball. **d-e**, Percent of MPOA^*Esr1*∩*Vgat*^ stimulation time mounting either a ball or a female for all trials (d) and for trials separated based on which object the mouse contacts first (e; *n* = 9 mice). **f**, Mean number of USVs in response to MPOA^*Esr1*∩*Vgat*^ stimulation prior to object contact or during contact. **g**, Schematic (left) and raster plots of run and bilateral reach head-fixed behaviors (top right) and mean number of USVs (bottom right) under conditions MPOA^*Esr1*∩*Vgat*^ stimulation alone, object presentation alone or both. *p > 0.05, ***p < 0.001; ns, not significant. For all figure panels, data are mean ± SEM. See Table S2 for further details of the statistical analyses.

Male mice sing to, mount and copulate with females. Recent work has identified neurons in the male medial preoptic area of the hypothalamus (MPOA^*Esr1*∩*Vgat*^; oestrogen receptor 1-expressing GABAergic neurons, Fig. 1a) that reliably induce these behaviours and are required for their natural expression^3,16,17^. Which behaviours are elicited depends on the context of MPOA^*Esr1*∩*Vgat*^ activation: singing (ultrasonic vocalizations (USVs)) occurs in the presence or absence of a target^13–15^, but the transition to mounting requires the presence of a target object^3,17^ (Fig. 1b,c and Extended Data Fig. 1a,b). To determine what object features are important for releasing mounting, we compared how optogenetic activation of MPOA^*Esr1*∩*Vgat*^ neurons elicited mating attempts towards conspecifics or mountable 3D inanimate target objects (that is, ‘dummy females’) in freely moving animals.

MPOA^*Esr1*∩*Vgat*^ optogenetic activation invariably induced time-locked mounting accompanied by singing (USV+ mount) of females, males and even mouse-sized 3D inanimate objects (ball or toy mouse; Fig. 1b,c, Extended Data Fig. 1c–e and Supplementary Videos 1 and 2). The indiscriminate nature of mountable targets (Fig. 1c) indicated that a common sensory feature derived from encountered 3D objects can trigger behavioural release. This feature required object-derived mechanosensory cues, but not visual cues (Fig. 1d). Evoked mounting was blocked by numbing the face, but it was not eliminated by whisker removal alone. USV+ mounting only occurred when MPOA^*Esr1*∩*Vgat*^ stimulation coincided with direct contact with an object, but not when stimulation was delivered on approach but terminated before contact (Fig. 1e and Extended Data Fig. 1f). Thus, MPOA^*Esr1*∩*Vgat*^ photostimulation induces a mating drive state that can be expressed as singing in solitary animals or as mounting on contact with a 3D target object.

## Sites integrating touch and sexual drive

We next investigated where and how the brain integrates target-derived mechanosensory cues with MPOA^*Esr1*∩*Vgat*^ inputs to release consummatory behaviours. To this end, we developed a custom robotic arm system to present head-fixed, awake-behaving animals with target objects (for example, a ball) with or without photostimulation of MPOA^*Esr1*∩*Vgat*^ neurons, while performing acute silicon probe extracellular recordings (Fig. 1f). Target presentation consisted of an approach, contact and retreat phase, in essence ‘animating’ an object.

During MPOA^*Esr1*∩*Vgat*^ stimulation, head-fixed mice performed many of the behaviours observed during free interactions. This included running and singing, which occurred in the absence of a target, and bilateral reaching that only occurred on contact with the target (Fig. 1g and Extended Data Fig. 1g). In naturalistic mating, bilateral reaching precedes mounting as males use both forelimbs to grasp a female to initiate a mount. The ability to perform mounting is restricted under head fixation, therefore we used USV+ bilateral reaching as a proxy for mount attempts. Indeed, when provided with ‘graspable’ targets, such as a toy mouse with fur, head-fixed mice during MPOA stimulation reached, grasped and pulled the objects towards them (Supplementary Video 3).

**Extended Data Fig. 2:**
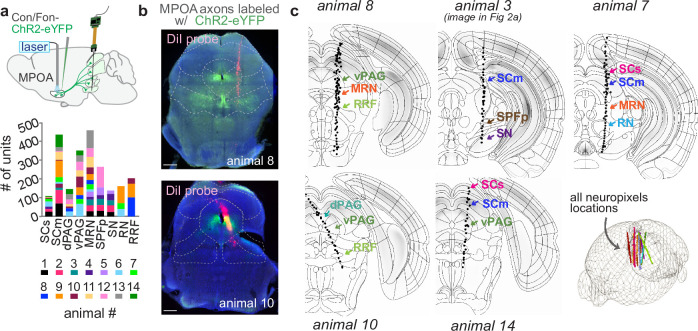
Locations of simultaneous recordings downstream of the MPOA. **a**, Experimental paradigm (top) and number of single units recorded across the nine regions (*n* = 14 mice, 1-2 sessions per mouse). **b**, Images of Neuropixels locations (DiI, pink) from animal 8 and 10. MPOA^*Esr1*∩*Vgat*^ axons are labeled in green. DAPI, blue. Scale; 0.5 mm. **c**, Representative electrode track reconstructions using the Allen common atlas space and locations of all probes in a 3D rendered brain. SCs, superior colliculus sensory-related; SCm, superior colliculus motor-related; dorsal periaqueductal grey (dPAG); ventral periaqueductal grey (vPAG); midbrain reticular nucleus, (MRN); subparafascicular nucleus (SPFp), substantia nigra (SN); red nucleus (RN); retrorubral field (RRF).

In these animals, we used high-density Neuropixels probes to acutely record simultaneously from regions in the midbrain and thalamus to which MPOA^*Esr1*∩*Vgat*^ neurons project and which are also known to receive mechanosensory inputs (Fig. 1h,i and Extended Data Fig. 2a–c). We recorded from nine regions including the sensory-related and motor-related superior colliculus (SCs and SCm, respectively), dorsal and ventral periaqueductal grey, midbrain reticular nucleus, parvocellular subparafascicular nucleus of the thalamus (SPFp), substantia nigra, red nucleus and the retrorubral field. Electrode tracks were labelled with a lipophilic dye (DiI) and reconstructed at the end of each experiment (Extended Data Fig. 2b,c).

**Extended Data Fig. 3:**
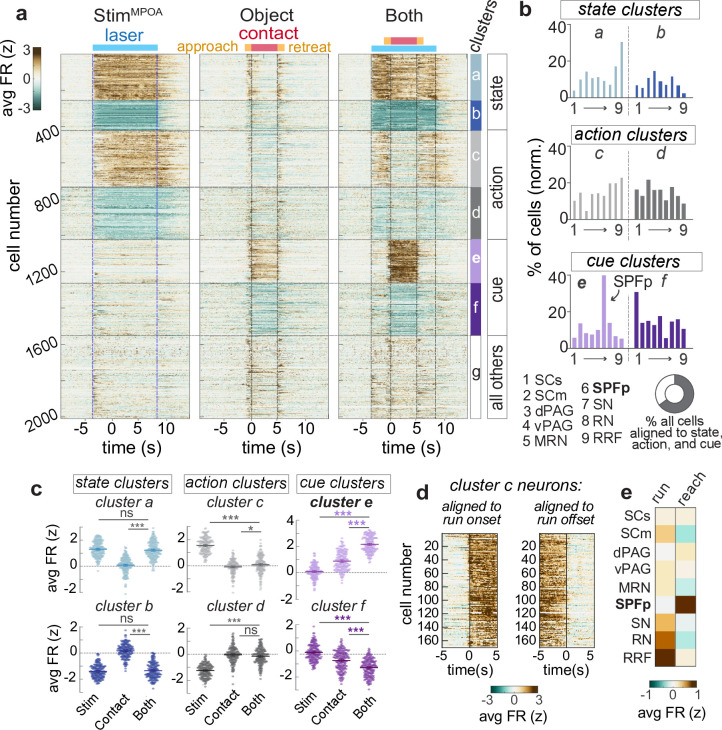
Distributed representations of cue, state and action representations across all recorded brain regions. **a**, Mean z-scored firing rate across trials for all recorded cells in the three conditions: MPOA stimulation alone (stim^MPOA^), object presentation alone (Contact) or both. Neurons are sorted by k-means clustering. **b**, Percent of cells in each cluster by brain region, normalized by the total cells recorded per region. Pie chart depicts total cells with time-locked activity to state (stim^MPOA^), action (run) and cue (object presentation). **c**, Mean activity for each cell per cluster in response to MPOA stimulation, object contact or both. **d**, Mean activity for cluster c neurons aligned to the onset and offset of running in MPOA stimulation conditions. **e**, Mean population firing rate across recorded brain regions during running or reaching in multimodal conditions. SCs, superior colliculus sensory-related; SCm, superior colliculus motor-related; dorsal periaqueductal grey (dPAG); ventral periaqueductal grey (vPAG); midbrain reticular nucleus, (MRN); subparafascicular nucleus (SPFp), substantia nigra (SN); red nucleus (RN); retrorubral field (RRF). *p < 0.05, ***p < 0.001; ns, not significant. For all figure panels, data are mean ± SEM. See Table S2 for further details of the statistical analyses.

Recordings were performed during three conditions: (1) MPOA photostimulation (stim^MPOA^) alone to elicit a sexual drive state; (2) target (dummy female) presentation alone to provide contact cues; and (3) multimodal (both) conditions with concurrent MPOA stimulation and target contact to trigger USV+ bilateral reaching (Fig. 1f,g). We recorded from 2,300 well-isolated single units (more than 100 units per region) from 14 animals (3 or more animals per region; Extended Data Fig. 2a). Across all regions, we observed neurons with changes in spiking activity that were time-locked to object contact (SCm example unit), MPOA stimulation (ventral periaqueductal grey example unit) and MPOA-elicited running (retrorubral field example unit; Fig. 1j and Extended Data Fig. 3a–d). These cue, state and action representations were broadly distributed across all regions and together comprised roughly 65% of all recorded neurons (Extended Data Fig. 3b,e).

To identify integration sites, we looked for neurons that were differentially modulated during multimodal conditions compared to that in the two unimodal conditions. In stark contrast to the broadly distributed responses to unimodal stimuli described above, neurons that showed significantly higher spiking activity in the multimodal condition were highly enriched in just one region, the SPFp (Fig. 1k). These neurons comprised roughly 40% of all recorded SPFp neurons, in contrast to only roughly 5–15% of neurons in all other regions. The overall mean activity of the SPFp was significantly higher in the multimodal condition compared to either condition alone (Fig. 1l). No other region showed this property. These cross-regional comparisons from simultaneously recorded neurons identified the SPFp as a prime candidate for performing sensory-state integration to release mounting.

## Nonlinear integration in the SPFp

**Fig. 2:**
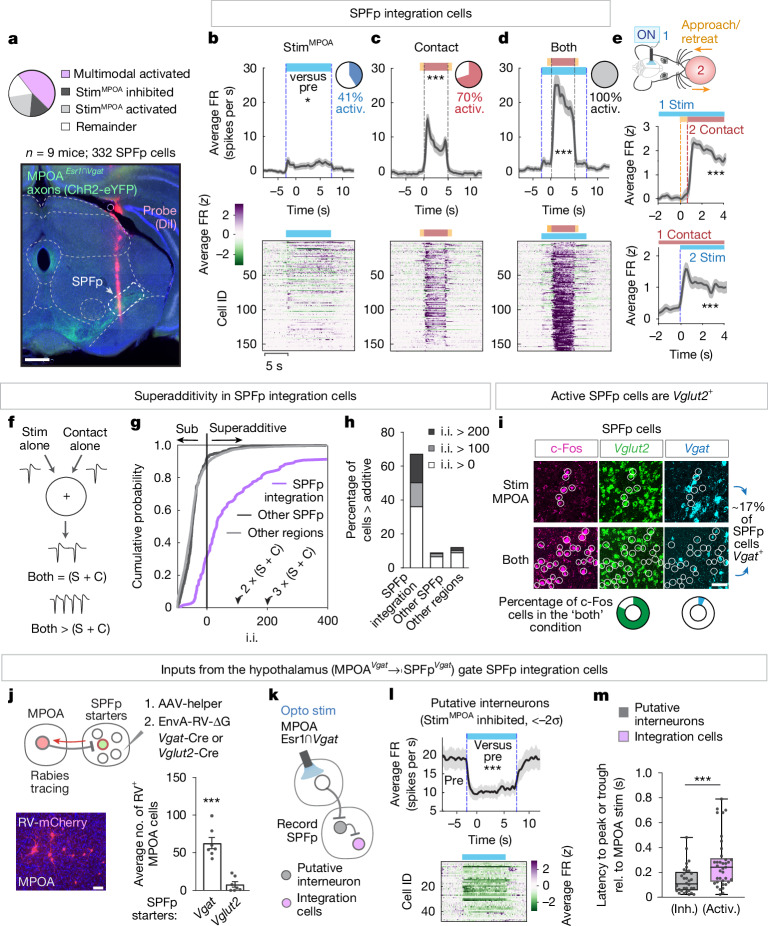
SPFp neurons nonlinearly integrate MPOA input and target-contact cues. **a**, Percentage of SPFp cells responding to MPOA stimulation (more than 2*σ* or less than −2*σ*; top). Representative image of recording location (DiI, pink; MPOA^*Esr1*∩*Vgat*^ axons, green; bottom). **b**–**d**, Normalized mean firing rate for each cell (*z *score, bottom) and the population of SPFp integration cells (spikes per s, top) during stim^MPOA^ alone (**b**), contact alone (**c**) or both (**d**; *n* = 159 cells, 9 mice; 10 trials per condition). Inset pie chart depicts the percentage of cells activated relative to baseline (more than 2*σ*). Cells in **b**–**d** were sorted in the same way. activ., activation. **e**, Normalized mean firing rate (*z *score) of integration cells when stim^MPOA^ precedes object contact (top) or the reverse (bottom; *n* = 52 cells, 4 mice; 10 trials per condition). **f**, Diagram depicting additive or superadditive interactions between MPOA stimulation (S) and object contact (C). **g**, Cumulative distribution of integration indices (i.i.; Methods). Integration cells in purple (*n* = 159 cells), all other SPFp cells in black (*n* = 173 cells) and all other cells from the other regions in grey (*n* = 1,807 cells). **h**, Percentage of cells with integration indices above (more than 0), double (more than 100) or triple the additive (more than 200). **i**, Representative image of *Vgat* and *Vglut2* SPFp cells labelled with c-Fos after multimodal or unimodal stimuli by FISH. **j**, Monosynaptic rabies tracing from SPFp *Vgat* or *Vglut2* starter cells (schematic, top). Representative image (bottom left) and mean number of labelled MPOA cells (bottom right; *n* = 7 *Vgat*, *n* = 7 *Vglut2* mice). **k**, Circuit schematic. **l**, Mean firing rate for each cell (*z *score, bottom) and the population of putative interneurons (spikes per s, top). **m**, Latency to peak for SPFp integration cells and putative interneurons (*n* = 40 interneuron, *n* = 73 integration cells, 4 mice). Inh., inhibition; rel., relative. *P < 0.05, ***P < 0.001. For all figure panels, data are mean ± s.e.m. Box plot (25th, median and 75th percentiles) and whiskers (minimum to maximum). See Supplementary Table 2 for further details of the statistical analyses. Scale bars, 0.5 mm (**a**) and 50 μm (**i**,**j**).

To address how SPFp performs sensory-state integration, we identified SPFp cells significantly activated in multimodal relative to unimodal conditions (more than two standard deviations (2*σ*); herein referred to as ‘integration’ cells) to determine their coding properties (Fig. 2a, top). Although the SPFp is strongly innervated by MPOA^*Esr1*∩*Vgat*^ axons (Fig. 2a, bottom), MPOA stimulation by itself evoked relatively weak responses at the population level (Fig. 2b, stim^MPOA^). Nonetheless, 41% of integration cells significantly increased their firing during MPOA stimulation relative to baseline (more than 2*σ*; Fig. 2b, pie chart). When presenting the object alone, a large fraction of these cells showed significantly increased activity during the object contact phase (roughly 70% of cells more than 2*σ* above baseline; Fig. 2c, pie chart). When MPOA stimulation coincided with object contact, SPFp integration cells showed robust, sustained increased activity, on average 20 spikes per s (all cells more than 2*σ* above baseline; Fig. 2d). Presentation order did not matter for the observed multimodal enhancement (Fig. 2e).

Superadditivity is a feature often observed in multimodal neurons, which is thought to enhance responses to behaviourally relevant, salient stimuli^22,23^. The key aspect of this feature is its nonlinearity: the responses to the two types of input do not sum linearly. To quantitatively distinguish additive from superadditive responses of SPFp cells, we constructed an integration index (Methods; Fig. 2f–h). Non-zero values of this index classify a cell as superadditive, meaning it shows a higher spiking rate when both stimuli are presented together (stim^MPOA^ + contact) compared to the sum of the response to each individual stimulus (stim^MPOA^ alone + contact alone). An index of 100 indicates a 2-fold (100%) increase over the additive response. On the basis of this index, most (roughly 70%) SPFp integration cells were classified as superadditive compared to only roughly 12% of all other cells located in the eight other recorded regions (Fig. 2g,h). As MPOA is thought to control mating motivation or drive^3,16,17,24^, this suggests that the superadditive responses could reflect a state-dependent enhancement of contact responses during mating.

Paradoxically MPOA^*Esr1*∩*Vgat*^ inputs, despite being GABAergic, exerted an overwhelmingly positive-acting influence on SPFp integration cell activity. Could this sign inversion be imparted through disinhibition, as has been shown in other targets of the MPOA^14,15^? If so, then MPOA inputs should project directly to SPFp inhibitory interneurons and not to integration cells. To address this, we first determined the neurotransmitter phenotype of integration cells by means of fluorescence in situ hybridization (FISH). Almost all multimodal active (stim^MPOA^ + contact) c-Fos^+^ cells were labelled by *Vglut2* suggesting that integration cells are excitatory (Fig. 2i). Second, we traced monosynaptic inputs to SPFp^*Vgat*^ inhibitory cells and, in a separate cohort, SPFp^*Vglut2*^ excitatory cells using Cre-dependent rabies tracing (Methods). This confirmed that SPFp^*Vgat*^ cells received significantly more inputs from the MPOA than did SPFp^*Vglut2*^ cells (Fig. 2j). This is suggestive of indirect, di-synaptic influences of MPOA onto SPFp integration cells (Fig. 2j).

In alignment with this disinhibition circuit hypothesis (MPOA^*Esr1*∩*Vgat*^ ⇥ SPFp^*Vgat*^ ⇥ SPFp^*Vglut2*^; Fig. 2k), our SPFp recordings did reveal a distinct population of neurons that was strongly inhibited by MPOA^*Esr1*∩*Vgat*^ photostimulation. These inhibited neurons showed high baseline firing (on average 20 spikes per s; Fig. 2l), which is characteristic of inhibitory interneurons in other brain regions. These cells comprised roughly 15% of all SPFp neurons recorded, consistent with the observed density of *Vgat*-expressing (*Vgat*^+^) neurons in SPFp (Fig. 2i) and interneurons in other thalamic nuclei^25,26^. If MPOA inputs function to disinhibit SPFp integration cells through SPFp interneurons, then activation of integration cells should lag behind that of inhibited cells following MPOA stimulation. Calculating the latency to peak (or trough) confirmed this with significantly shorter latencies for inhibited cells than integration cells (Fig. 2m).

## Coding of socially relevant contact

**Fig. 3:**
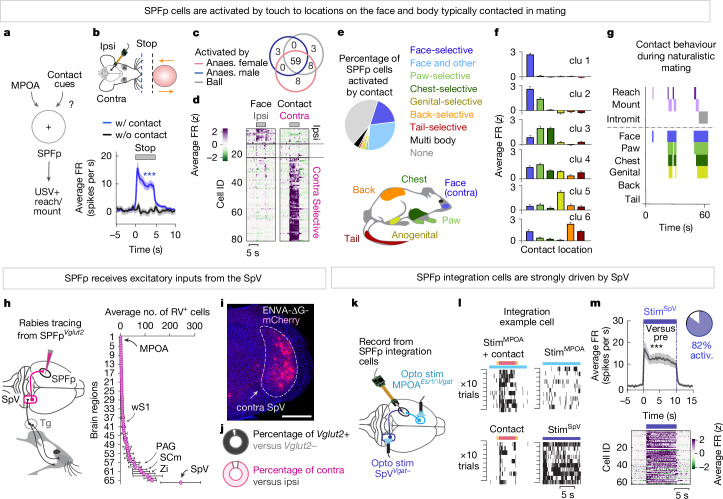
Excitatory inputs from SpV relays target-contact cues to SPFp. **a**, Diagram depicting inputs to SPFp. **b**, Normalized mean firing rate of SPFp integration cells to objects presented with (w/) versus without (w/o) contact (*n* = 142 cells, 7 mice; 10 trials per condition). Contra, contralateral; ipsi, ipsilateral. **c**, Percentage of SPFp integration cells significantly activated by contact with a ball or anaesthetized (Anaes.) conspecific. **d**, Mean firing rate of SPFp integration cells to objects presented to the ipsilateral or contralateral face (*n* = 82 cells, 4 mice; 10 trials per condition). **e**, Percentage of SPFp cells activated by contact to different parts of the body. **f**, Mean firing rate (*z *score) of face-responsive neurons clustered into non-overlapping groups using *k*-means (Methods). **g**, Raster plot of contact behaviours towards a female in a freely behaving mating encounter. The plot shows the body sites contacted during naturalistic mating. **h**, Whole-brain cell counts from tracing SPFp^*Vglut2*^ inputs (*n* = 3 mice). Tg, trigeminal ganglion. **i**, Representative image of mCherry^+^ retrogradely labelled cells in the SpV. **j**, Relative contralateral versus ipsilateral (bottom; *n* = 4 mice) and *Vglut2*^+^ versus *Vglut2*^−^ (top; *n* = 3 mice) retrogradely labelled cells in SpV. **k**, Schematic of recording strategy. **l**, Spike rasters for a SPFp integration cells across the four conditions. **m**, Normalized mean firing rate for each cell (*z* score, bottom) and the population of SPFp integration cells (spikes per s, top) in response to SpV^*Vgat*−^ optogenetic stimulation (*n* = 62 cells, 4 mice; 10 trials per condition). Inset pie chart depicts the percentage of cells activated by SpV^*Vgat*−^ stimulation relative to baseline (more than 2*σ*). ***P < 0.001. For all figure panels, data are mean ± s.e.m. See Supplementary Table 2 for further details of the statistical analyses. Scale bar, 0.5 mm.

**Extended Data Fig. 4:**
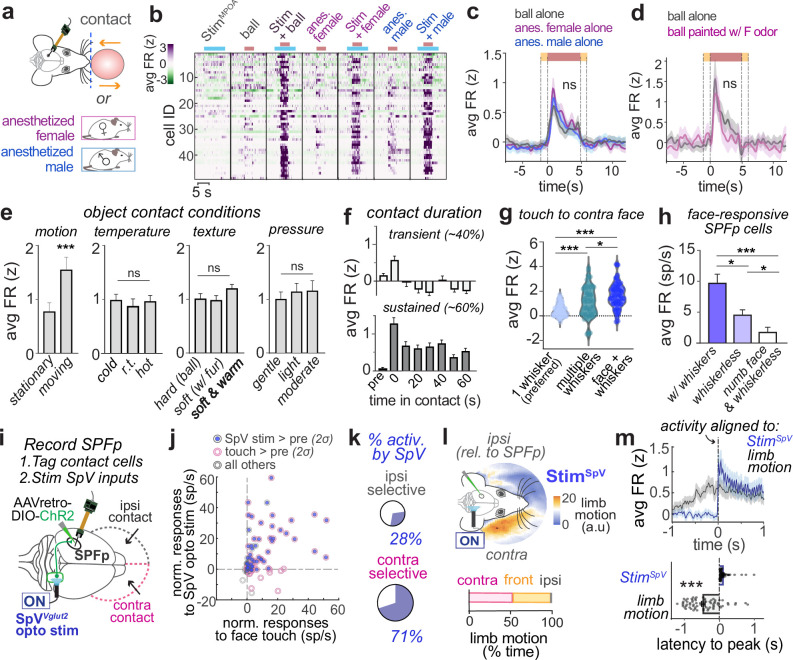
Sensory features encoded by the SPFp. **a**, Schematic of experimental paradigm. **b-c**, Mean firing rate for each SPFp integration cell (b) and the population (c) in response to a ball or anesthetized conspecifics (*n* = 49 cells, 3 mice; 10 trials per condition). **d**, Same as in c, but for a ball painted with female urine to provide pheromonal odors. **e**, Mean activity of SPFp cells to various object contact conditions by the robotic arm. **f**, Mean activity to object contact over time of transient (top; *n* = 35 cells) and sustained (bottom; *n* = 50 cells) SPFp face-responsive cells. Bin size; 2 s. **g**, Mean activity of face-responsive SPFp cells to touch to one whisker (most preferred for each cell), multiple whiskers or entire contra face (*n* = 81 cells; 3 mice). **h**, Mean activity of face-responsive SPFp cells to object contact under whiskers cuts and face numb conditions (*n* = 71 cells; 3 mice). **i**, Schematic of recording strategy. **j**, Normalized mean firing rate of all SPFp cells to face touch and SpV^*Vglut2*→SPFp^ stimulation (*n* = 94 cells; 4 mice; 10 trials per condition). **k**, Percent of SPFp cells activated by SpV^*Vglut2*→SPFp^ stimulation relative to baseline (> 2σ; choice probability >0.7 contra selective or <0.3 ipsi selective). **l**, Representative image of limb motion tracking (arbitrary units, top) and percent of limb movements (bottom) located ipsilateral, in front, and contralateral relative to the SPFp during SpV^*Vglut2*→SPFp^ stimulation. **m**, Mean activity of SPFp cells (top) and peak latency (bottom) aligned to SpV^*Vglut2*→SPFp^ stimulation (blue) or limb movement onset (grey; *n* = 94 cells). Dots represent individual cells. *p < 0.05, ***p < 0.001; ns, not significant. For all figure panels, data are mean ± SEM. See Table S2 for further details of the statistical analyses.

SPFp integration cells are disinhibited by hypothalamic sexual drive, but what types of sensory cues activate these neurons (Fig. 3a)? As most SPFp integration cells were weakly active during the contact phase of object presentation (Fig. 2c), we first asked whether non-contact object cues are sufficient to drive activity in these cells. Using the robotic arm, we varied the object distance and identity while performing electrophysiological recordings from SPFp integration cells. We first presented objects with the same approach and retreat phase but stopped the object at a short distance in front of the mouse (Fig. 3b, top). Integration cells were not significantly modulated by these no-contact presentations, suggesting that contact cues were the predominant determinant of activity (Fig. 3b, bottom). These cells had largely similar (that is, contact-dependent) responses to the presentation of various object types, including anaesthetized females, anaesthetized males, inanimate objects and objects painted with female urine (Fig. 3c and Extended Data Fig. 4a–e). These contact-induced responses were enhanced by presenting moving objects as compared to stationary ones (Extended Data Fig. 4e). Although some cells responded transiently at the moment of contact, most showed elevated firing lasting for minutes throughout the duration of contact (Extended Data Fig. 4f). This suggests SPFp cells are relatively simple object ‘contact detectors’, whose responses at the population level are enhanced by object motion during contact.

We next asked whether contact with any location on the body is able to evoke activity in SPFp cells, or only contact with specific areas. In a separate cohort of animals, we varied the site of object contact with different parts of the body, including the face, paw, chest, anogenital, back and tail regions. Almost all mechanosensory SPFp cells were significantly activated by touch to the face (Fig. 3d,e). However, more than half of these face-responsive cells also responded to touch to locations of the body typically contacted together in mating (face, paw, chest, anogenital regions; face and body group; Fig. 3e–g). Whereas some face-responsive cells responded weakly to touch delivered to an individual whisker, almost all responded robustly when touch was delivered to larger regions including several whiskers or the entire face (Extended Data Fig. 4g). These responses were effectively abolished on whisker removal and face numbing (Extended Data Fig. 4h). Face-responsive cells were mostly selective for touch delivered to the contralateral, but not ipsilateral face, suggestive of predominantly contralateral mechanosensory inputs (Fig. 3d).

Next, to uncover the source of mechanosensory inputs, we performed whole-brain rabies tracing to map the inputs to SPFp^*Vglut2*^ cells (Fig. 3h and Supplementary Table 1). We observed the most numerous inputs from the contralateral spinal trigeminal nucleus (SpV), the first central relay that receives face-derived mechanosensory input directly from peripheral sensory neurons in the trigeminal ganglion^27–29^ (Fig. 3h–j). These SpV inputs to SPFp were almost exclusively glutamatergic as determined by FISH (Fig. 3j). As SpV projection neurons are excitatory, we asked whether they activate SPFp integration cells. To address this, we optogenetically activated MPOA or SpV while we recorded from the SPFp (Fig. 3k). We first identified cells that showed multimodal responses to MPOA stimulation plus object contact (example unit in Fig. 3l), and then examined their responses when SpV glutamatergic neurons (genetically accessed as *Vgat*-negative cells; Methods) were activated. SPFp integration cells were strongly driven by SpV stimulation in a highly time-locked manner, with 82% of cells significantly activated (more than 2*σ* above baseline; Fig. 3m). SpV stimulation also resulted in the selective activation of contralateral but not ipsilateral face contact-tuned cells (Extended Data Fig. 4i–k).

**Extended Data Fig. 5:**
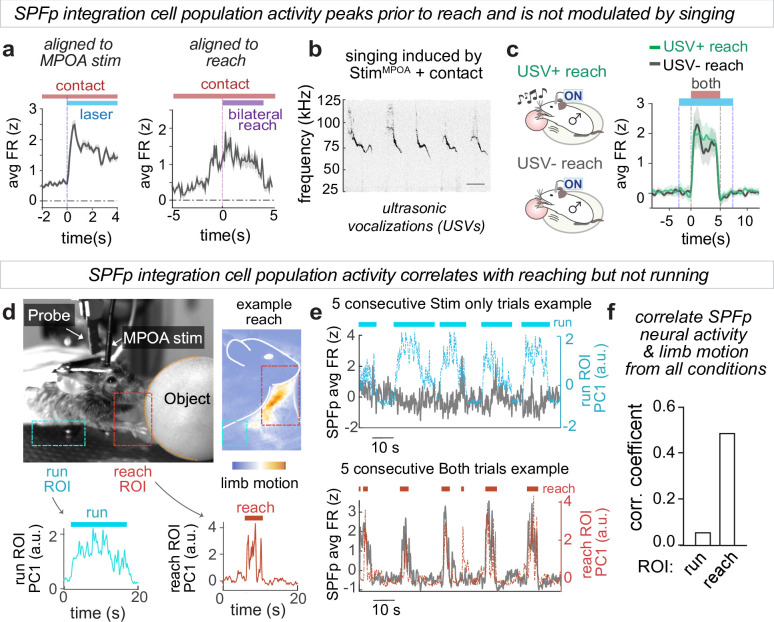
SPFp neural activity correlates with reaching, but not running. **a**, Mean activity of SPFp cells in multimodal (stim^MPOA^ + contact) conditions aligned to MPOA stimulation onset (left) or bilateral reach onset (right; *n* = 52 cells, 4 mice; 10 trials per condition). **b**, Representative spectrogram of evoked singing (ultrasonic vocalizations, USVs). Scale, 100 ms. **c**, Mean activity of SPFp cells during trials with and without singing. **d**, Representative tracking of limb motion in two ROIs during running and reaching. **e**, Representative overlay of aligned SPFp cell activity and limb motion during running (top) or reaching (bottom). f, Correlation of SPFp cell activity and limb motion. ns, not significant. For all figure panels, data are mean ± SEM.

In the absence of a target object, SpV photostimulation often evoked time-locked forelimb movements, as if something was touching the animal’s face (Extended Data Fig. 4l and Supplementary Video 4). This raised the possibility that the observed changes in SPFp activity may be a consequence of elicited motor behaviours. Yet this was not the case, as the evoked activity in SPFp was tightly coupled to SpV stimulation onset (within tens of milliseconds) and preceded any overt motor action (Extended Data Fig. 4m). The peak activity of SPFp integration cells also preceded motor actions (that is, reach initiation) in multimodal conditions (stim^MPOA^ + contact; Extended Data Fig. 5a). This activity was not further modulated by the presence of USVs (Extended Data Fig. 5b,c). Whereas the overall activity of SPFp integration cells was elevated during moments of USV+ bilateral reaching (which occurred during multimodal stimuli), it was not elevated during moments of running suggesting it is not reflective of generalized arousal or motion (Extended Data Fig. 5d–f). Thus, although SPFp activity could reflect motor planning or a propensity to reach (that is, a releasing cue), it is probably not a consequence of sensory-motor feedback associated with the reach itself.

## *Pth2* marks multimodal SPFp neurons

**Fig. 4:**
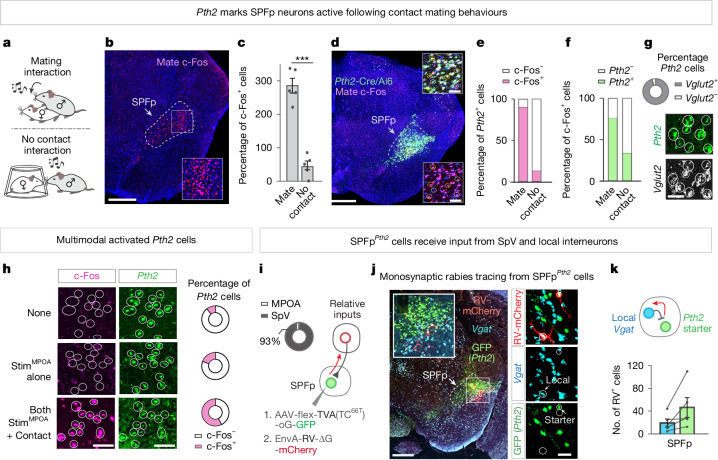
*Pth2* marks mating-activated multimodal SPFp neurons. **a**, Experimental model for mating and no-contact interactions. **b**, Representative image of c-Fos^+^ neurons (pink) in SPFp following mating. **c**, Mean number of c-Fos^+^ SPFp cells following mating and no-contact control conditions (*n* = 5 mate, *n* = 5 control mice). **d**, Representative image of *Pth2*^+^ (green; *Pth2*-Cre/Ai6 mouse) and c-Fos^+^ neurons following mating. Insets, high magnification images from SPFp. **e**,**f**, Percentage of *Pth2*^+^ cells labelled with c-Fos (**e**) or the reverse (**f**) following mating and control conditions (*n* = 3 mate, *n* = 3 control mice). **g**, Quantification (top) and representative image (bottom) of *Pth2*^+^ (green) and *Vglut2*^+^ (white) neurons by FISH. **h**, Quantification (right) and representative images (left) of SPFp^*Pth2*^ cells labelled with c-Fos after multimodal (stim^MPOA^ + contact), unimodal (stim^MPOA^) or no stimulation conditions by FISH. **i**, Schematic of monosynaptic rabies tracing from SPFp^*Pth2*^ starter cells (right). Relative number of mCherry^+^ cells labelled in the MPOA and SpV (left; *n* = 4 mice). **j**, Representative image from a *Pth2*-Cre mouse with SPFp cells labelled with TVA-oG-GFP (green), RV-mCherry (red) and *Vgat* (blue) by FISH. High magnification in the right panel. **k**, Number of mCherry-labelled SPFp *Pth2* starter cells and local *Vgat* cells (*n* = 5 mice; 4 sections per mouse). ***P < 0.001. For all figure panels, data are mean ± s.e.m. See Supplementary Table 2 for further details of the statistical analyses. Scale bars, 0.5 mm (**b**,**d**,**j** (left)), 100 μm (**h**) and 50 μm (**d** (inset),**g**,**j **(right)).

Our head-fixed electrophysiological recordings demonstrated that many SPFp neurons are activated by contact with an object in a manner dependent on a sexual drive state provided by concurrent MPOA stimulation. This raised the question whether and how these SPFp neurons function in freely behaving males during naturalistic mating interactions. To address this, we first used the activity marker c-Fos to determine whether the SPFp is active following either copulation with a female or, as a control, after the presentation of a female protected by a pencil cup to allow a no-contact interaction (Fig. 4a). Following copulation, dense labelling of c-Fos^+^ neurons was observed in the SPFp, whereas far fewer c-Fos^+^ cells were observed in the no-contact control (Fig. 4b,c).

We then sought to identify a genetic marker for SPFp mating-active neurons to perturb and record calcium signals from these cells. The neuropeptide parathyroid hormone 2 (*Pth2*), also known as tuberoinfundibular peptide of 39 residues (TIP39), has previously been shown using c-Fos to label SPFp neurons activated during male reproductive behaviours^30–34^, but the dynamics of this activation and its causal role in mating are unknown. We therefore asked whether SPFp mating-active neurons express *Pth2*, using a *Pth2*-Cre driver. Crossing this driver with a Cre-dependent Ai6 reporter resulted in highly restricted labelling of SPFp neurons in a location that matched our electrophysiological recording sites (Fig. 4d, compare Fig. 2a). Following mating, roughly 90% of SPFp *Pth2*-Cre-expressing (Ai6^+^) neurons were labelled by c-Fos, whereas only roughly 10% were labelled in controls with no-contact female exposure (Fig. 4e). Moreover, most c-Fos^+^ SPFp mating-active neurons were labelled by *Pth2* (Fig. 4f). FISH revealed that these SPFp *Pth2*-expressing neurons were glutamatergic (Fig. 4g). Also, a large fraction of these SPFp^*Pth2*^ neurons were active following multimodal (stim^MPOA^ + contact) conditions as compared to baseline control conditions in freely behaving animals (Fig. 4h).

If *Pth2* marks SPFp integration cells, then they would be expected to receive direct inputs from SpV excitatory neurons and indirect, di-synaptic inputs from MPOA inhibitory neurons. To test this prediction, we used EnvA-pseudotyped rabies to trace monosynaptic inputs to SPFp^*Pth2*^ cells expressing TVA^66T^ (lower affinity mutant version of the EnvA receptor)^35,36^. First, we compared the relative number of monosynaptically labelled mCherry input cells in the MPOA and SpV. There were almost no MPOA inputs but many SpV inputs to these cells, as expected for integration cells (Fig. 4i, donut plot). Second, we asked whether *Pth2*^+^ cells receive monosynaptic inputs from local inhibitory interneurons (that is, SPFp^*Vgat*^ ⇥ SPFp^*Pth2*^). To identify these cells, we performed triple FISH in the SPFp to label local *Vgat*^+^ cells, TVA-expressing (*Pth2*^+^) cells (by means of expression of GFP) and RV-mCherry cells. We observed rabies-mCherry labelling of *Vgat*^+^, *Pth2*^−^ (GFP^−^) cells in the vicinity of *Vgat*^−^, *Pth2*^+^ (GFP^+^) starter cells (Fig. 4j,k), suggesting that SPFp^*Pth2*^ cells receive monosynaptic inputs from local interneurons.

Taken together, these data support the existence of a di-synaptic pathway from MPOA^*Vgat*^ ⇥ SPFp^*Vgat*^ ⇥ SPFp^*Pth2*^. MPOA *Vgat*^+^ neurons directly project to and can inhibit SPFp local inhibitory neurons (Fig. 2j), which in turn presumably inhibit SPFp *Pth2*^+^ cells, resulting in disinhibition of the latter when MPOA is activated. This identified circuit motif aligns with our electrophysiological observations and suggests that SPFp integration cells express *Pth2*.

## SPFp^*Pth2*^ activity is critical for mating

**Fig. 5:**
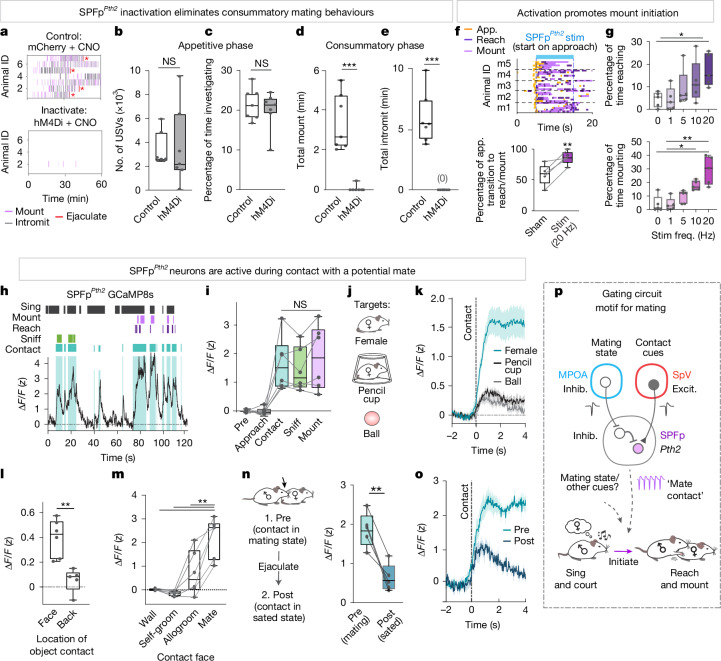
SPFp^*Pth2*^ neurons encode social contact and causally control consummatory mating. **a**, Raster plots of male mating behaviours towards females following CNO in mice injected with hM4Di or control mice. **b**–**e**, Inactivation of SPFp^*Pth2*^ neurons during mating. **b**, Number of USVs. **c**, Percentage of time investigating females. **d**, Time spent mounting. **e**, Time spent intromitting (*n* = 7 hM4Di, *n* = 7 control mice). **f**,**g**, Optogenetic activation of SPFp^*Pth2*^ neurons on approach to a female during mating (that is, pseudo-closed-loop). **f**, Raster plots (top) of behaviours following stimulation. Percentage of approach trials that transition to reach and/or mount (bottom). m1–m5 denote mouse 1–mouse 5. **g**, Percentage of stimulation time spent reaching (top) or mounting (bottom) for sham, 1, 5, 10 and 20 Hz frequency stimulation (*n* = 5 ChR2 mice; six trials per condition). **h**, Representative scaled calcium signals (GCaMP8s) from SPFp^*Pth2*^ neurons during exposure to a female. **i**, Mean activity of SPFp^*Pth2*^ neurons during no-contact and contact behaviours with a female (*n* = 6 mice). **j**, Experimental model. **k**, SPFp^*Pth2*^ neuron activity aligned to contact onset with an object, a female under a pencil cup or a female in a free interaction (*n* = 6 mice). **l**, Mean activity during face or back object contact (*n* = 6 mice). **m**, Same, but for contact with the walls of the cage, during self-groom, allogrooming and mating. **n**,**o**, Mean activity during contact (**n**) and activity aligned to contact (**o**) with a female before ejaculation (mating state) or immediately after ejaculation (sated state; *n* = 6 mice). **p**, Summary schematic of findings. Dots on bar plots in **b**–**g** and **i**–**n** represent data from individual animals. *P < 0.05, **P < 0.01, ***P < 0.001; NS, not significant. For all figure panels, data are mean ± s.e.m. **b**–**g**,**i**,**l**–**n**, Box plot (25th, median and 75th percentiles) and whiskers (minimum to maximum). Excit., excitation; Inhib., inhibition. See Supplementary Table 2 for further details of the statistical analyses.

**Extended Data Fig. 6:**
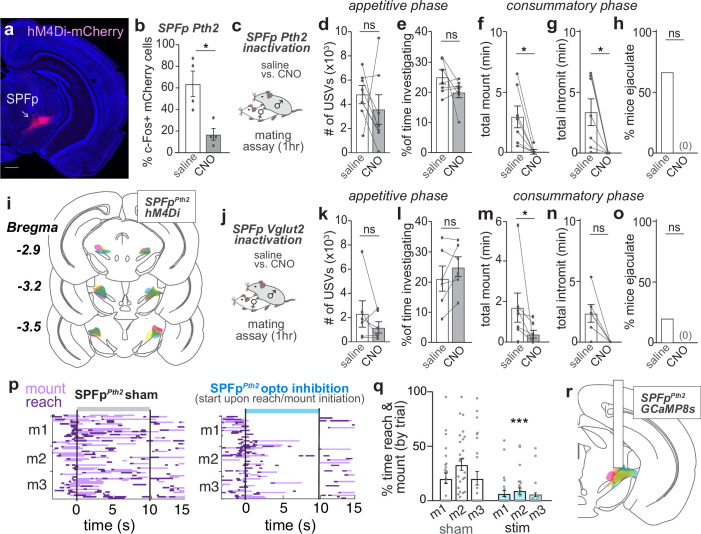
SPFp neurons are required for consummatory but not appetitive phases of mating. **a**, Representative image of hM4Di-mCherry expression in SPFp^*Pth2*^ neurons (pink). Scale; 0.5 mm. **b**, Percent of hM4Di-mCherry+ cells labeled with c-Fos from mice injected with saline or CNO and presented with females (*n* = 4 mice hM4Di/saline, *n* = 4 mice hM4Di/CNO). **c**, Strategy to inactivate SPFp^*Pth2*^ neurons. **d-h**, Within animal comparison (CNO versus saline) of SPFp^*Pth2*^ neurons inactivation during mating. (d) Number of USVs. (e) Percent of time investigating females. (f) Time spent mounting. (g) Time spent intromitting. (h) Percent of mice that ejaculate (*n* = 7 hM4Di). **i**, Locations of hM4D-mCherry expressing cells. Color indicates individual animals. **j**, Strategy to inactivate SPFp^*Vglut2*^ neurons. **k-o**, Within animal comparison (CNO versus saline) of SPFp^*Vglut2*^ neurons inactivation during mating. (k) Number of USVs. (l) Percent of time investigating females. (m) Time spent mounting. (n) Time spent intromitting. (o) Percent of mice that ejaculate (*n* = 6 hM4Di). **p**, Raster plots of SPFp^*Pth2*^ optogenetic inhibition (right) or sham (left) conditions during mating behaviors towards females (*n* = 3 stGtACR2 mice, 20 trials per mouse). **q**, Percent of time reaching and mounting for stimulation and sham conditions by trial. **r**, Locations of GCaMP8s expressing cells. Dots on bar plots in (d-g), and (k-n) and colors in plots (i,r) represent data from individual animals.*p < 0.05, ***p < 0.001 ; ns, not significant. For all figure panels, data are mean ± SEM. See Table S2 for further details of the statistical analyses.

Having established using c-Fos labelling that SPFp^*Pth2*^ neurons are active following copulation, we next asked whether these neurons play a causal role in male reproductive behaviours. To address this, we expressed either hM4Di-mCherry or mCherry (control) in SPFp *Pth2*-Cre neurons using Cre-dependent adeno-associated virus (AAV) vectors. Following clozapine *N*-oxide (CNO) injections, males were paired with a hormonally primed receptive female for 1 hour (Fig. 5a). Control mice spent most of the time performing mating behaviours including singing, investigating, mounting and intromission (Fig. 5b–e). CNO-injected mice expressing hM4Di in SPFp^*Pth2*^ neurons showed similar amounts of singing and investigating relative to controls, suggesting that their inhibition did not disrupt mating drive or social interest (Fig. 5b,c). In stark contrast to controls, however, SPFp^*Pth2*^ neuron inhibition resulted in an almost complete elimination of consummatory mating actions (Fig. 5d,e). Only one out of seven hM4D mice showed mounting and none showed intromission or ejaculation (Fig. 5a, raster plots), whereas all controls showed mounting and intromission and roughly 60% achieved ejaculation. Perturbations of all glutamatergic SPFp neurons also resulted in a similar trend of reduced consummatory behaviours, as did within-animal comparisons of saline versus CNO conditions and optogenetic inhibition of SPFp^*Pth2*^ neurons (Extended Data Fig. 6a–q). Thus, although SPFp^*Pth2*^ neural activity was not required for appetitive behaviours, it was critical for performing contact-mediated consummatory actions during mating encounters.

**Extended Data Fig. 7:**
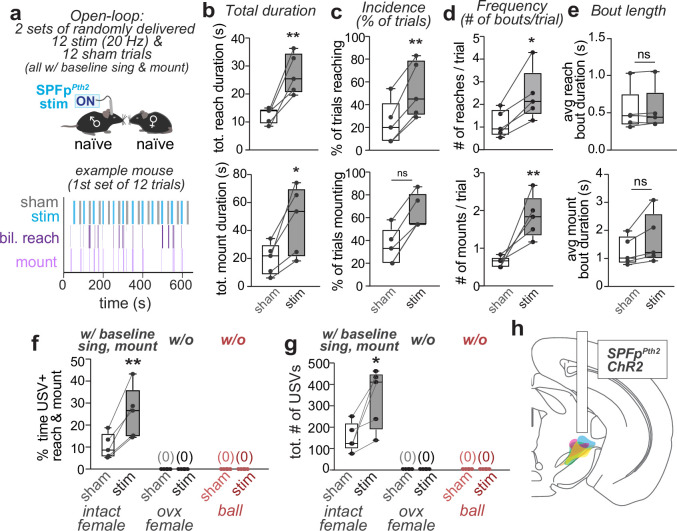
SPFp^*Pth2*^ neurons promote the initiation of consummatory behaviors in an ongoing mating interaction. **a**, Experimental design (top) and example behavior raster (bottom) for randomized stimulation or sham trials with an intact female (i.e. open-loop). **b-e**, Reaching (top) and mounting (bottom) during randomized optogenetic stimulation or sham trials. (b) Total duration. (c) Incidence, percent of trials. (d) Frequency, number of bouts per trial. (e) Mean bout length (*n* = 5 mice). **f**, Percent of SPFp^*Pth2*^ optogenetic stimulation or sham time spent mounting an intact female (all with baseline mounting), unprimed ovariectomized female (ovx; subset of trials without baseline mounting) or ball. **g**, Same as f, but for number of USVs. **h**, Locations of ChR2-eYFP expressing cells. Dots on bar plots in (b-g) and colors in plots (h) represent data from individual animals. *p < 0.05, **p < 0.01; ns, not significant. For all figure panels, data are mean ± SEM. Box plot (25^th^, median and 75^th^ percentiles) and whiskers (min to max). See Table S2 for further details of the statistical analyses.

To determine whether SPFp^*Pth2*^ neurons are sufficient for releasing consummatory behaviours, we optogenetically activated these cells in males as they approached females (that is, closed-loop; Fig. 5f, top). SPFp^*Pth2*^ activation significantly increased the probability of an approach transitioning into bilateral reaching and/or mounting compared to matched sham control trials (Fig. 5f, bottom). Increased reaching and mounting occurred when the stimulation frequency was high (10 Hz or 20 Hz) but not when it was low (1 Hz or 5 Hz; Fig. 5g). This increase was due to a higher incidence of reach and mount initiation as opposed to the prolongation of individual behavioural bouts, as determined by randomized stimulation trials (that is, open-loop; Extended Data Fig. 7a–e). Only animals that showed spontaneous mounting during baseline responded to stimulation (Extended Data Fig. 7f–h). Activation never promoted mounting of inanimate objects or, in some cases, even of ovariectomized unprimed females that otherwise did not evoke singing or mounting from investigating males. This observation suggests that SPFp^*Pth2*^ neurons do not appear to elicit a sexual drive state, but rather promote the release of consummatory behaviours when the drive state is present, consistent with the above loss-of-function results.

We next explored the dynamics of SPFp^*Pth2*^ neuronal activation in males during mating encounters to determine which phases of the social interaction and what object features most strongly activated these cells in freely behaving animals. To answer these questions, we performed fibre photometry to monitor bulk-calcium signals in SPFp^*Pth2*^ cells expressing GCaMP8s (Fig. 5h and Extended Data Fig. 6r) during an interaction with a sexually receptive female. Peaks of SPFp^*Pth2*^ activity coincided with the onset of female contact, but not with the onset of mounting or bilateral reaching (although the latter behaviours occurred while activity was elevated; Fig. 5h,i). Notably, SPFp^*Pth2*^ neural activity was significantly elevated in a time-locked manner during all moments of contact regardless of the behaviour performed and was not modulated by no-contact behaviours such as singing or approach (Fig. 5h,i).

We then examined SPFp^*Pth2*^ neuron activity as the animals contacted various objects (Fig. 5j). SPFp^*Pth2*^ neurons showed significantly higher responses during interactions with females (a roughly eightfold increase) relative to interactions with inanimate objects or no-contact interactions with females under a pencil cup (Fig. 5j,k). This increase in activity was observed during object contact with the face, but not with the back (Fig. 5l). Activity was also elevated in some animals during allogrooming in which males groomed a female, but importantly not during self-grooming behaviours (Fig. 5m). Nevertheless, this activity induced by contacting a female during non-sexual allogrooming was significantly lower than that during sexual interactions.

These observations raised the question of whether contact-mediated activation of SPFp^*Pth2*^ neurons in freely behaving males was dependent on a mating drive state. Because it was not possible (due to interference between two different Cre drivers) to combine silencing of MPOA^*Esr1*∩*Vgat*^ neurons with calcium recording in SPFp^*Pth2*^ neurons, to investigate this question we took advantage of the observation that following ejaculation males enter a period of sexual satiety in which their mating drive is significantly reduced^37–39^. Recovery from this period typically takes several hours to days. We therefore compared the activation of SPFp^*Pth2*^ neurons by contact cues in males before versus after ejaculation. Notably, contact with a female before ejaculation (that is, in a mating drive state) resulted in significantly higher SPFp^*Pth2*^ activity relative to contact with that same female just after ejaculation (that is, in the sated state) (Fig. 5n,o). Together, these data indicate that SPFp^*Pth2*^ neuronal activity encodes contact cues in a manner that depends on the internal mating drive state of the animal, and that this activity is essential for successful copulation.

## Discussion

Here we describe a mechanism that governs the transition from the appetitive to the consummatory phase of male mating, a crucial but relatively under-studied step in reproductive behaviour^40–42^. Our results indicate that this transition is controlled by a gate that operates at moments of contact with a potential mating partner. We discovered that SPFp^*Pth2*^ neurons perform this gating function by implementing an algorithm that combines a temporally imprecise sexual arousal state (by means of MPOA disinhibitory input) with a temporally precise mechanosensory cue (by means of SpV excitatory input) in a nonlinear manner, to encode moments of social contact (Fig. 5p). The output from this gate may be decoded by a downstream thresholding mechanism to allow grasping and mounting throughout a mating encounter. This thresholding may include the release (at high spiking rates) of *Pth2* itself, a neuropeptide that has been shown to be regulated by social contact in zebrafish^43^.

Although our silencing experiments indicated that SPFp^*Pth2*^ activity is required for all consummatory behaviours, fibre photometry indicated that their activity is time-locked to moments of contact with the mating partner, not to specific actions such as mounting. This suggests that SPFp^*Pth2*^ neurons function as mate- or social-contact detectors that gate the initiation of mounting towards an appropriate target. As in sensorimotor systems, separating the gate that permits and initiates a behaviour from the circuit that executes it helps filter noise and support flexible, adaptive responses^44,45^.

The effects of activating SPFp^*Pth2*^ neurons support this interpretation. Optogenetic stimulation of these cells increased the frequency of reach and mount initiation towards females but did not prolong individual behavioural bouts. However, de novo mounting of inanimate objects was not evoked. The latter suggests either that optogenetic SPFp^*Pth2*^ stimulation cannot surpass the activation threshold for mounting in the absence of mating drive (perhaps due to strong tonic inhibition by local interneurons), or that SPFp output itself may require further integration with mating drive downstream. In both cases, a contingent requirement for mating drive would prevent every contact that activates SPFp from automatically eliciting mounting towards any object. This layered control may reflect the need for many checkpoints to execute an action sequence that requires sustained contact with an actively moving target.

Classical ethological studies have shown that the release of innate social behaviours in many animals often requires species and/or sex-specific ‘sign stimuli’ detected by the visual system^4,5,46^. In this system the releasing cue is neither specific nor visual. Instead, object contact cues seem generic and specificity is provided by the drive state encoded by MPOA^*Esr1*∩*Vgat*^ neurons, presumably in response to pheromonal cues^47^. This may explain why hypothalamic stimulation can evoke behaviours towards inanimate objects devoid of species or sex-specific identification cues^3,17,18^. Whether similar contact-driven gating mechanisms support other social behaviours, such as aggression or parenting, remains to be determined.

Beyond its role in male sexual functions described in this study, the SPFp (also known as the posterior intralaminar nucleus, PIL) has recently been implicated in maternal care and affiliative grooming in females. This points to a potentially broader role of this thalamic region in mediating social touch^48–50^. Given the importance of touch in social and sexual functions, including bonding, reward and social or sexual satiety^51–56^, our findings open new avenues to investigate how social touch is encoded in the brain, and how it is integrated with internal states to guide adaptive social behaviours.

Our physiological studies suggest that mechanosensory responses in SPFp are evoked by generic object contact with the face and/or with other body parts (responses to which are enhanced by stimulus movement). A distinct population of SPFp^CGRP^ neurons detects threats by encoding multimodal innately aversive or noxious stimuli, including foot shock, predator odour and visual looming stimuli^32,57^. Together, these data suggest that the SPFp may harbour anatomically and molecularly distinct subpopulations that encode low-dimensional but valence-specific representations of salient sensory cues, which are integrated with drive state to trigger rapid adaptive responses.

Taken together, our results identify a computation, an algorithm and its circuit-level implementation that control the transition from the appetitive to the consummatory phase of male reproductive behaviour. The interactive synergy between hypothalamic and mechanosensory inputs in SPFp can imbue meaning to a generic target, transforming it into an object of desire and thereby initiating the coordination and patterning of consummatory mating actions. The logic uncovered here may generalize to appetitive to consummatory transitions underlying other social and goal-directed behaviours^6,58,59^. Our findings also open avenues to understanding the function of a highly conserved neuropeptide^31,34,43,48^ in the control of mating behaviour, which in turn may have translational implications for treatments of sexual dysfunction in humans^60^.

## Methods

### Animals

All experimental procedures were carried out in accordance with National Institutes of Health (NIH) guidelines and approved by the Institute Animal Care and Use Committee and the Institute Biosafety Committee at the California Institute of Technology (Caltech). Mice were housed in ventilated micro-isolator cages in a temperature- and humidity-controlled environment under a reverse 12-h light/dark cycle. Food and water were provided ad libitum. Mouse cages were changed weekly. *Esr1*^*flp*^ knock-in mice (JAX no. 036028), *Slc32a1(Vgat)*^*cre*^ knock-in mice (Jax no. 028862), *Slc17a6(Vglut2)*^*cre*^ knock-in mice (Jax no. 028863), *Ai6* (Jax no. 007906), were backcrossed into the C57BL/6N background (more than N10) and bred at Caltech. BALB/c female mice, ovariectomized and intact, were used as intruder mice and purchased from Jackson Laboratory. *Pth2*^*cre*^ knock-in mice were bred at Caltech and purchased from GemPharmatech (stock no. T054004). Heterozygous *Vglut2*^*cre*^, *Pth2*^*cre*^ or double heterozygotes *Esr1*^*flp*^, *Vgat*^*cre*^, *Pth2*^*cre*^ *Ai6* mice were used for cell-specific targeting experiments and were genotyped by PCR analysis using genomic DNA from tail or ear tissue. Adult mice were 8–12 weeks old at the time of viral injection. Animals were randomly assigned to different experimental conditions. Group sample sizes were chosen on the basis of previous studies.

### Viruses

AAVDJ-hSyn-Con/Fon-hChR2(H134R)-eYFP and AAV2-syn-Flex-jGCaMP8s were packaged at the HHMI Janelia Research Campus virus facility. AAVRetro-EF1a-DIO-hChR2(H134R)-eYFP, AAVDJ-hSyn-Con/Fon-eYFP, AAV2-hSyn-DIO-hM4D-mCherry, AAV2-hSyn-DIO-mCherry, AAV8-EF1a-Nuc-flox(mCherry)-EGFP and AAV1-hSyn1-SIO-stGtACR2-FusionRed were purchased from Addgene. AAV9-EF1a-DO-hChR2(H134R)-mCherry, AAV8-DIO-TC66T-2A-eGFP-2A-oG, G-Deleted Rabies-mCherry and EnvA G-Deleted Rabies-mCherry were purchased from Salk Institute. AAVDJ-hSyn-DIO-ChR2(H134R)-eYFP was purchased from the UNC Vector Core. ‘Con/Fon’ indicates Cre-ON/Flp-ON virus; ‘DO’ indicates Cre-OFF virus. All virus titres were 10^12^ or more genomic copies per millilitre.

### Stereotaxic surgery

Surgeries were performed on adult mice aged 8–12 weeks. Mice were anaesthetized with 1–3.0% isoflurane and placed into a stereotaxic alignment system (David Kopf Instruments). A small craniotomy hole was drilled with a dental drill. Virus (0.1–0.3 μl; titre, 10^12^ particles per ml) was then injected into the target area using a pulled glass capillary (World Precision Instruments) and a pressure injector (Micro4 controller, World Precision Instruments), at a flow rate of 20 nl min^−1^. The capillary was then slowly retracted at least 5 min after infusion. Stereotaxic injection coordinates were based on the Paxinos and Franklin atlas (MPOA, bregma −0.1, midline ±0.5, dorsal surface −4.7; SPFp bregma −3.1, midline ±1.5, dorsal surface −3.7; SpV bregma −6.5, midline ±1.8 and dorsal surface −5.2). For optogenetic and fibre photometry experiments, fibre optic cannulas (optogenetics: diameter 200 μm, numerical aperture 0.22; fibre photometry: diameter 400 μm, numerical aperture 0.5; Thorlabs) were subsequently placed 300–500 μm above the virus injection sites and fixed on the skull with dental cement (Parkell). Mice were allowed to recover for at least 2 weeks before behavioural testing. Half of the mice in each cage were randomly assigned to either treatment or control groups. All control mice were treated with the same experimental procedures, but a control virus was injected instead.

For acute electrophysiological recordings combined with optogenetics, viral injections were performed and an implanted fibre optic cannula and a steel headplate was fixed to the skull with dental cement. One day before electrophysiological recordings, animals were briefly anaesthetized and a 32 AWG chlorinated silver wire (A-M system) with a presoldered gold pin was implanted through a small hole and cemented to the skull to provide chronic grounding. One or more craniotomies (less than 1 mm in diameter) were drilled and then covered with a drop of silicone oil (30,000 cSt, Aldrich) followed by a silicone sealant (KwikCast, World Precision Instruments) until the experiment was performed. Animals were single housed following surgery and allowed to recover before recordings.

For MPOA^*Esr1*∩*Vgat*^ optogenetic recording experiments, viruses encoding intersectional^61,62^ (Cre and Flp)-dependent channelrhodopsin (Con/Fon-ChR2-eYFP) was injected unilaterally into the MPOA, ipsilateral to the recording site in *Esr1*^*flp*^*;Vgat*^*cre*^ mice. For MPOA^*Esr1*∩*Vgat*^ and SpV^*Vgat*−^ dual optogenetic experiments, Con/Fon-ChR2-eYFP was injected into the ipsilateral MPOA and Cre-OFF ChR2 (DO-ChR2-mCherry) was injected into the contralateral SpV in *Esr1*^*flp*^*;Vgat*^*cre*^ mice. For SpV^*Vglut2*−>SPFp^ optogenetic recording experiments, a retrogradely labelling Cre-dependent virus (AAVRetro-DIO-ChR2-eYFP) was injected into the SPFp and the fibre optic was implanted over the contralateral SpV in *Vglut2*^*cre*^ mice. For SPFp^*Pth2*^ optogenetic experiments, Cre-dependent ChR2 (activation) or stGtACR2 (inactivation) was injected bilaterally into the SPFp in *Pth2*^*cre*^ mice. For SPFp^*Pth2*^ chemogenetic experiments, Cre-dependent hM4Di was injected bilaterally into the SPFp in *Pth2*^*cre*^ mice. For SPFp^*Pth2*^ fibre photometry experiments, Cre-dependent GCaMP8s^63^ was injected unilaterally into the SPFp in *Pth2*^*cre*^ mice.

For monosynaptic rabies tracing, viruses encoding Cre-dependent mutated TVA (TC66T) and rabies G glycoprotein was injected into the SPFp of *Vgat*^*cre*^, *Vglut2*^*cre*^ or *Pth2*^*cre*^ animals^35^. Two weeks later, EnvA G-Deleted Rabies-mCherry was injected into the same location in the SPFp^36^. Mice were housed in a biosafety room for 4–6 days to allow the rabies virus to infect and express mCherry in presynaptic cells before the brains were harvested.

### Electrophysiological recordings

Mice were head-fixed, the silicone sealant was removed and physiological saline was applied to the skull to cover the craniotomy. Electrophysiological recordings were made using Neuropixels 1.0 probes, recording from the 384 most distal channels^64^. The reference and the ground contacts on the Neuropixels probes were permanently soldered together. Recordings were made using an external reference configuration achieved by connecting the probe reference to the chronically implanted silver wire on the skull. To allow post hoc tracking of probe trajectories, the electrode shank was coated with DiI (Invitrogen). The Neuropixels probe was attached to an aluminium dovetail adaptor screwed to an aluminium rod and lowered with a micromanipulator (Sensapex) at roughly 2 μm s^−1^ until reaching the target depth. Data acquisition began 20 min after reaching the final depth. Neural signals were acquired at 30 kHz using Open Ephys software^65^. For optogenetic stimulation, a fibre optic cannula was connected to a blue 470-nm laser (Shanghai Laser and Optics Century). Before data acquisition, the laser was turned on to check for a photoelectric artefact in the neural recording. Animals in which the laser power that reliably induce behaviours was too high and, therefore, produced a photoelectric artefact were not included. After each recording experiment, probes were slowly retracted and immersed in 1% Tergazyme solution to remove tissue and silicone oil residues.

For the initial large-scale screen, we recorded from a total of 2,300 single units from 9 nine brain regions across 14 mice. This included 110 units in SCs, 442 units in SCm, 148 units in dorsal periaqueductal grey, 356 units in numerical aperture ventral periaqueductal grey, 465 units in midbrain reticular nucleus, 268 units from SPFp, 140 units from the substantia nigra, 164 units from the red nucleus and 207 single units from the retrorubral field. Including this screen, we recorded from a total of 837 single units from the SPFp across 18 mice (11 with MPOA and/or SpV stimulation, seven without stimulation).

### Optogenetic stimulation

Mice with fibre optic implants were connected to an optic fibre (200-μm diameter, numerical aperture 0.22; Doric Lenses) and allowed to habituate before behavioural testing. The optic fibre was connected to a 470-nm laser to deliver blue light (Shanghai Laser and Optics Century). Before behavioural testing, the light intensity achieved at the tip of the optic fibre was estimated by connecting an equivalent optic fibre to the patch cable and measuring the light intensity at the tip of the fibre using a power meter. Laser power was controlled by turning an analogue knob on the laser power supply. The laser was either triggered manually when animals were engaged in a behaviour of interest or automatically in a series using a Pulse Pal (Open Ephys). For optogenetic stimulation in freely behaving and head-fixed animals, mice were given trains of photostimulation (10-ms pulse, 20 Hz for 10 s). Sham stimulation (laser off) was interleaved as an internal control. For freely moving SPFp^*Pth2*^ optogenetic experiments, animals were given a 5-min baseline interaction period. Then, 2 sets of 12 photostimulation and 12 interleaved sham stimulation trials were delivered every 30 s using a Pulse Pal. This ensured randomized stimulation periods throughout the interaction. Frequency titration experiments involved series of 1, 5, 10 and 20 Hz pulse trains delivered by the experimenter on approach of the female.

### Chemogenetic inhibition

Mice were injected with hM4Di-mCherry^66^ or mCherry (control)-expressing AAVs in the MPOA of *Pth2*^*cre*^ mice. Males were sexually naive and single housed 1 day before behavioural testing. Behavioural tests were performed with at least 1 day in between tests. Mice were intraperitoneally injected with CNO (Enzo; 5.0 mg kg^−1^) dissolved in saline 40 min before behavioural testing. All animals received CNO or saline on the same day with the experimenter blind to the treatment group.

### Fibre photometry

To measure bulk florescence, mice with fibre optic implants were connected to an optical fibre (400-μm diameter, 0.5 numerical aperture; Thorlabs) to both deliver excitation of light and collect emitted florescence. We used 470-nm light-emitting diodes (LEDs) (M470F3, Thorlabs, filtered with 470–10-nm bandpass filters FB470-10, Thorlabs) for fluorophore excitation and 405-nm LEDs for isosbestic control (M405FP1, Thorlabs, filtered with 410–10-nm bandpass filters FB410-10, Thorlabs). Each excitation wavelength (470 and 405 nm) was sinusoidally modulated at a distinct carrier frequency (208 and 333 Hz, respectively) that is demodulated to recover the original calcium sensor response (that is, lock-in amplification)^67^. This modulation step minimizes contamination of the calcium signal by changes in overall ambient light and low-frequency noise. The emission signal from the 470-nm excitation was normalized to the emission signal from the isosbestic excitation (405 nm), to control for motion artefacts, photobleaching and levels of GCaMP8 expression. LEDs were coupled to a 425-nm longpass dichroic mirror (Thorlabs, DMLP425R) by means of fibre optic patch cables (diameter 400 μm, numerical aperture 0.5; Thorlabs). Emitted light was collected through the patch cable, coupled to a 490-nm longpass dichroic mirror (DMLP490R, Thorlabs), filtered (FF01-542/27-25, Semrock), collimated through a focusing lens (F671SMA-405, Thorlabs) and detected by the photodetectors (model no. 2151, Newport). Recordings were acquired using Synapse software (Tucker Davis Technologies).

Mice were habituated to the optic fibre cable before behavioural testing. For behaviour testing, mice were presented with an inanimate object (ball), a hormonally primed female under a pencil cup and then free access to a hormonally primed female. All data analyses were performed in MATLAB. Behavioural videos, audio and fibre photometry data were time-locked. *F*_n_ was calculated using normalized (405 nm) fluorescence signals from 470-nm excitation. *F*_n_(*t*) = 100 × (*F*_470_(*t*) − *F*_405fit_(*t*)/*F*_405fit_(*t*)). For *z-*scored data, traces were *z-*score normalized before averaging.

### Histology

Verification of virus expression, implant placement and Neuropixels probes (DiI) were performed on all mice. Mice lacking correct viral expression or probe targeting were excluded from analysis. Mice were transcardially perfused with saline, followed by 4% paraformaldehyde in 1× PBS. Brains were collected and postfixed overnight, then cryoprotected in 30% sucrose. Brains were embedded in optimal cutting temperature mounting media, frozen on dry ice and sectioned coronally at 40–100 μm on a cryostat (Leico Biosystems). Sections were immunolabeled and counterstained with 4,6-diamidino-2-phenylindole (0.5 μg ml^−1^) washed and mounted onto slides. Primary antibodies were rabbit-anti-DsRed (1:1,000, Takara Bio 632496) and goat anti-Fos (1:500, Santa Cruz, sc52-g). Secondary antibodies were Alexa Fluor 594 donkey anti-rabbit (1:1,000, Invitrogen, A-21207) and Alexa Fluor 647 donkey anti-goat (1:1,000, Invitrogen, A-21447). FISH (RNAScope, ACD Bio) was performed with probes targeting *Pth2* (1052361), *mCherry* (431201), *GFP* (409011), *SLC32a1* (*VGAT*, 319191) and *SLC17a6* (*VGLUT2*, 319171) following the manufacturer’s protocol. Sections were imaged with an epifluorescent (Olympus VS120) or a confocal microscope (Leica).

### Behavioural assays

#### Head-fixed behaviours during acute recordings

Object presentation was controlled by a robotic arm (ufactory, uArm) that allowed for the selection of *xyz* coordinates, speed and duration of presentation. Top and side infrared cameras (Basler, acquired at 10 Hz) were used to record behaviours and triggered in Python. Audio recordings were collected at a 250-kHz sampling rate using an Avisoft-UltraSoundGate116H kit with a condenser ultrasound microphone CM16/CMPA (Avisoft-Bioacoustics). Laser stimulation was triggered by a Pulse Pal (Open Ephys) that was time-locked to the robotic arm controlled in Python. All video, audio, laser and robotic arm TTL signals were concurrently acquired on a NIDAQ (National Instruments) and later aligned to neural recordings in MATLAB.

Object trial bouts involved presentations of an object (ping pong ball, toy mouse and so on) that approached a mouse, maintained position for 5 s and then retracted. Laser trial bouts involved 10 s of 10-ms pulses at 20 Hz. Multimodal trial bouts involved either laser on first and after a 2-s delay the object was presented or the reverse. Interstimulus intervals were at least 5 s within a bout and several minutes between trial bouts. Trial bouts (object, laser and multimodal) were randomly interleaved. For presentation of conspecifics, BALB/c females or males were briefly anaesthetized and presented with the anogenital region towards the face of the recorded mouse. For presentation of objects with female odours, urine from the anogenital region of a BALB/c female mouse was swabbed onto a ball to be immediately presented using the robotic arm. The location of object contact was varied to touch different regions of the body, lasting 5 s for each trial. For object features, the same object was presented that varied in temperature, textures or robot-controlled pressure.

#### Behavioural monitoring

All behavioural experiments were performed during the animals’ dark cycle. Mice were habituated in the room for 10–20 min before testing. Behavioural tests were performed in conventional mouse housing cage (home cage or new cage) using the previously described behaviour recording set up^68^. For optogenetic experiments, most behaviour tests were performed under white light. All other tests were performed under red light. Both top and front cameras (forward-looking infrared, Grasshopper) acquired video at 30 Hz using StreamPix7 (Norpix). Audio recordings were collected at a 250-kHz sampling rate using an Avisoft-UltraSoundGate116H kit with a condenser ultrasound microphone CM16/CMPA (Avisoft-Bioacoustics) that was positioned 45 cm above the cage. Video and audio recordings were synchronized through a signal generated by StreamPix7.

#### Hormone priming

To enhance the sexual receptivity of female mice, hormone primed ovariectomized BALB/c female mice were used as stimulus animals in some experiments (chemogenetic, Fos, fibre photometry). Females were injected subcutaneous with 10 μg of β-estradiol-3-benzoate (E8515, Sigma-Aldrich) in sesame oil (S3547, Sigma-Aldrich) at 48 h, and 500 μg progesterone (P0130, Sigma-Aldrich) at 4–6 h before behavioural testing^3,69^. Females were tested before the assay for receptivity with a stud male mouse until up to three mounting attempts occurred, and any that demonstrated rejection behaviours were not used.

#### Mating assay

Male mice were single housed before testing. On the test day, males were acclimated in his home cage under the recording set up. A sexually experienced BALB/c female mouse was then introduced into the male’s cage either for free interaction or under a pencil cup (controls). For MPOA optogenetic experiments, animals were given 20 min to interact with a female or an object (ball) with randomly interleaved laser and sham stimulation. For sensory testing conditions, male mice received optogenetic stimulation with an object under white light, infrared light, following whisker trimming and whiskerless with topical lidocaine applied to the face in that order. For c-Fos induction, animals were allowed to interact until ejaculation and then perfused 1 h after ejaculation or 1 h after time matched exposures to a female under a pencil cup (controls). For chemogenetic and fibre photometry experiments, animals were given 1 h to interact with a female. At the end of hM4Di experiments, animals were either given saline or CNO and allowed to interact with a female for 1 h to induce c-Fos. To reduce the baseline mounting for SPFp^*Pth2*^ optogenetic experiments, sexually naive males (never ejaculated) were used and paired with either sexually naive C57BL/6N or ovariectomized unprimed BALB/c females. None of these interactions proceeded into intromission as the females rejected most mount and intromission attempts. This allowed for shorter but more frequent re-initiation of reach and mount attempts.

### Data analysis

#### Behaviour annotations

Behaviour videos (collected at 30 Hz) of mating interactions were first processed using a custom automated behaviour classifier system (the Mouse Action Recognition System, MARS) to generate frame-by-frame annotations of mounting and investigation behaviour^70^. Classifier annotation output, videos and spectrograms of recorded audio were then loaded into a custom, MATLAB-based behaviour annotation interface and classifier annotations were manually corrected by trained individuals blind to the experimental design. For head-fixed behaviours, videos were manually annotated. Final annotations include investigate (sniff), approach, chase, bilateral reach, mount, intromission and ejaculation. Bilateral reaching was defined as moments reaching for a conspecific or object when both of the male mouse’s forelimbs were off the ground. Mounting was defined as moments when both forelimbs were off the ground and the chest of the animal was on top of the conspecific or object. Intromission was defined as moments when the animal intromits, characterized by slow thrusting. Ejaculation was defined as the moment just preceding the male mouse falling over. Mount, reach or intromission bouts were considered terminated when all four limbs of the male mouse were on the ground, either when the male disengaged or when the female ran away. Thus, reaching and mounts during the pursuit of a female results in a series of shorter bouts whenever the distance between them exceeds the length of the animal’s forelimbs.

#### USV detection

USV audio files were saved as in the 16-bit WAV format and later analysed with DeepSqueak v.3.0, a deep learning-based software for detection and analysis of USVs^71^. The built-in mouse call detecting network was used to identify USV syllables and then corrected by manual inspection. Detection files were then exported and analysed in MATLAB. The number of USVs as a function of time was calculated and aligned with video and neural recordings.

#### Motion tracking

Videos of head-fixed mice acquired at 10 frames per second, were cropped to regions of interest (ROIs). ROIs corresponded to the region just below the mouse’s body to track limb motion during running (run ROI) and the region just in front of the chest to track limb motion during reaching (reach ROI). ROIs were converted to motion energy (change in pixel intensity from frame to frame) and the top 500 principal components were extracted for each ROI by singular value decomposition using FaceMap^72^. The first principal component of the motion data was used to correlate to the mean smoothed neural activity trace to obtain an *r* value.

#### Preprocessing and spike sorting

Neural signals from electrophysiological recordings were preprocessed by subtracting the median calculated within each group of 24 channels from the data to eliminate common-mode noise. The median subtracted data were spike sorted using Kilosort2.5 that in addition to the group median subtraction applied a high-pass filter (150 Hz), followed by whitening in blocks of 32 channels^73^. The cluster automatically labelled by Kilosort algorithm as ‘good’ were in turn manually curated by hand and further analysed with Phy2.

#### Firing rates

Spikes for each neuron were binned at 100-ms resolution and binned counts were divided by the bin width. For latency analysis, spikes were instead binned at 10-ms resolution. For some analyses, the rates were *z-*scored across the duration of the session per neuron. Baselines were the average number of spikes for a given 4 s before stimulus onset. Activated and inhibited cells were defined as responses at least 2*σ* from baseline. For clustering analysis, the *z-*scored trial average rates (20 s trials, 200 bins) were concatenated by neuron and *k*-means clustering was performed.

#### Selectivity index and choice probability

The selectivity index for each neuron was computed based on the average firing rate to two conditions (contralateral versus ipsilateral touch). The index was based on previous studies and is defined as: (response_contra_ − response_ipsi_)/(response_contra_ + response_ipsi_). Units with a high selectivity index (greater than 0.3) were considered contralateral selective. Units with low selectivity index (less than −0.3) were considered ipsilateral selective. These units were then selected for further analysis.

The choice probability of each neuron was computed as in previous work. Choice probability estimates the accuracy with which two conditions can be distinguished given the activity of each neuron. The choice probability of a given neuron for a pair of conditions (contralateral versus ipsilateral) is found by constructing a histogram of the activity of that cell (*F*(*t*)) under each of a selected pair of conditions and plotting the histograms against each other to generate a receiver operating characteristic curve. The area under this receiver operating characteristic curve is then computed by integration to generate the choice probability value for each unit with respect to each of the two conditions. This choice probability value is bounded from 0 to 1, with a choice probability of 0.5 indicating that the activity of the neuron cannot distinguish between the two conditions. Values greater than 0.7 or less than 0.3 were selected for further analyses.

#### Integration index

The integration index was computed for each neuron and was based on the average firing rate in a 5-s window for each condition (object only, MPOA stimulation only and multimodal). The integration index was computed based on previous studies: (response_Multi_ − response_additive_)/(response_additive_) × 100, where response_additive_ = (response_Monly_ + response_Oonly_). Units with an integration index above zero were considered superadditive integration cells and selected for further analysis.

#### Anatomical registration of electrode tracks

Electrode tracks were traced using DiI in coronal sections and registered to the Allen Mouse Brain Common Coordinate Framework^74^. Alignment was performed using SHARCQ to morph the atlas based on any asymmetries in the coronal slice and register locations of fluorescence^75^. Electrode locations were mapped using the Allen Mouse Brain Common Coordinate Framework and then cross-checked with the Franklin–Paxinos atlas and manual inspection. All single unit data used in the study were tagged with a corresponding location to the brain ROI. Units outside these anatomical boundaries were not included.

#### Cell counts

A brain map was overlain on the digital image to identify the appropriate regions using landmark structures, ventricles and optic tract as reference guides. An experimenter blind to condition analysed the images in ImageJ. For cell counts, the number of labelled cells were analysing using the automated ImageJ cell counter.

### Statistical analysis

Data were processed and analysed using Python, MATLAB and GraphPad (GraphPad PRISM v.9). The sample sizes were chosen on the basis of common practice in animal behaviour experiments. No statistical methods were used to predetermine sample size. Data were first tested for normality using a Shapiro–Wilk test and tested for homogeneity of variance using a Levene’s test. If data met normality and homogeneity of variance assumptions, parametric tests were used (for example, Student’s *t*-test, one-way or repeated-measures analysis of variance with Tukey’s multiple comparison). If not, non-parametric tests were used (for example, Mann–Whitney *U-*test or Kruskal–Wallis with Dunn’s multiple comparison test). Paired tests were used to compare within-group repeated-measures data (for example, Wilcoxon signed rank test and a Friedman test with Dunn’s multiple comparison). All statistical tests were two-sided. Significance levels are indicated as follows: *P < 0.05; **P < 0.01 and ***P < 0.001. For all representative images, similar results were obtained in at least three independent experiments.

### Reporting summary

Further information on research design is available in the Nature Portfolio Reporting Summary linked to this article.

## Online content

Any methods, additional references, Nature Portfolio reporting summaries, source data, extended data, supplementary information, acknowledgements, peer review information; details of author contributions and competing interests; and statements of data and code availability are available at 10.1038/s41586-025-09327-x.

## Supplementary information

Supplementary Information Supplementary Tables 1 and 2.

Reporting Summary

Supplementary Video 1 Optogenetic stimulation of MPOA^*Esr1*∩*Vgat*^ neurons triggers mounting towards a female. The ChR2-expressing male mouse was photostimulated while presented with a BALB/c female mouse. Stimulation period is indicated with ‘Laser ON’ and the LED light at the bottom right corner.

Supplementary Video 2 Optogenetic stimulation of MPOA^*Esr1*∩*Vgat*^ neurons triggers mounting towards a ball. The ChR2-expressing male mouse was photostimulated while presented with a ball. Stimulation period is indicated with ‘Laser ON’ and the LED light at the bottom right corner.

Supplementary Video 3 MPOA^*Esr1*∩*Vgat*^ optogenetic stimulation during acute Neuropixels recordings. Recordings were performed in the SPFp during MPOA^*Esr1*∩*Vgat*^ stimulation, object presentation or both.

Supplementary Video 4 SpV optogenetic stimulation during acute Neuropixels recordings. Recordings were performed in the SPFp during SpV^Vglut2−>SPFp^ stimulation.

## Data Availability

The data that support the findings of this study are available from the corresponding author upon request.
