# Supplementary material for: A circuit that integrates drive state and social contact to gate mating
Source: Nature. 2025 Sep 3;646(8084):394–403. doi: 10.1038/s41586-025-09327-x (PMC12507686; doi:10.1038/s41586-025-09327-x)
Supplement: Supplementary file 1 — Supplementary Tables 1 and 2. [file 41586_2025_9327_MOESM1_ESM.pdf]

nature portfolio

https://doi.org/10.1038/s41586-025-09327-x

Supplementary information

A circuit that integrates drive state and social contact to gate mating

In the format provided by the authors and unedited

Brain Region	Avg counts
Dorsomedial nucleus of the hypothalamus	1
Medial preoptic area	1
Retrosplenial area ventral part	1
Facial motor nucleus	1
Superior colliculus optic layer	1
Sublaterodorsal nucleus	1
Subthalamic nucleus	1.5
Superior central nucleus raphe	1.5
Parafascicular nucleus	1.5
Ventromedial hypothalamic nucleus	2
Nucleus raphe pallidus	2
Posterior hypothalamic nucleus	2
Endopiriform nucleus dorsal part	2.5
Dentate gyrus molecular layer	2.5
Substantia innominata	2.5
Laterodorsal tegmental nucleus	3
Anterior cingulate area dorsal part	3
Koelliker-Fuse subnucleus	3.5
Ventral posterolateral nucleus of the thalamus	3.5
Pontine central gray	3.5
Medial amygdalar nucleus	4
Parasubthalamic nucleus	4
Principal sensory nucleus of the trigeminal	4
Interposed nucleus	4.5
Dorsal nucleus raphe	4.5
Reticular nucleus of the thalamus	5
Inferior olivary complex	5
Bed nuclei of the stria terminalis	5
Piriform area	5
Magnocellular nucleus	5
Anterior amygdalar area	5
Central amygdalar nucleus	5.5
Lateral vestibular nucleus	5.5
Pallidum	6
Cuneiform nucleus	6.5
Nucleus raphe magnus	6.5
Supratrigeminal nucleus	7
Primary somatosensory area barrel field	7
Globus pallidus external segment	7
Striatum	7.5
Lateral hypothalamic area	8
Primary somatosensory area mouth	10
Primary somatosensory area upper limb	10.5
Nucleus prepositus	11
Pedunculopontine nucleus	11
Spinal vestibular nucleus	11.5
Superior vestibular nucleus	13
Fields of Forel	13
Pontine reticular nucleus	15.5
Supplemental somatosensory area	17
Paragigantocellular reticular nucleus	17
Pons	17
Intermediate reticular nucleus	23
Magnocellular reticular nucleus	26.5
Secondary motor area	28
Parabrachial nucleus	28
Periaqueductal gray	29
Medial vestibular nucleus	31.5
Pontine reticular nucleus caudal part	44
Superior colliculus motor related	44
Primary motor area	56.5
Zona incerta	62
Inferior colliculus	74
Caudoputamen	93
Gigantocellular reticular nucleus	113
**Spinal trigeminal nucleus**	**221.5**
**SPFp starter cells**	**335.3333333**
**SPFp local cells (non-starter)**	**143.6666667**

Supplementary Table 1: Whole-brain rabies tracing from the SPFp

Figure	Panel	Sample size	Test	Exact P-value, F-value with degreee of freedom for ANOVAs, t-vaule with degree of freedom for t-tests
1	c	n = 8 mice, 6 trials per condition	Friedman test	Friedman test: female condiition p=0.0001 (***). Dunn's multiple comparisons, p=0.0015 (**, stim only vs. both), p=0.0489 (*, female only vs. both). Male condiition p=0.0005 (***). Dunn's multiple comparisons, p=0.0054 (**, stim only vs. both), p=0.0054 (**, male only vs. both). Ball condiition p=0.0005 (***). Dunn's multiple comparisons, p=0.0054 (**, stim only vs. both), p=0.0054 (**, male only vs. both).
	d	n = 5 mice, 20 trials per mouse	One-way ANOVA with Tukey's multiple comparisons test	One way-ANOVA test: F(3, 16)=8.110, p=0.0009. Tukey's multiple comparisons test: p=0.6350 (light vs. dark), p=0.6350 (light vs. light-w), p=0.0014 (light vs. light-w+numb).
	k	n = 2300 total cells from 14 mice; 10 trials per condition	Each cell was considered activated or inhibited if mean firing rate during multimodal trials was >2σ or <-2σ (respecitvely) relative to both unimodal condition.	
	l	n = 2300 total cells from 14 mice; 10 trials per condition		
		n = 143 cells, SPFp	Friedman test	Friedman test: p<0.0001 (****). Dunn's multiple comparisons, p=0.8615 (ns, stim vs. contact), p<0.0001 (****, stim vs. both), p=<0.0001 (****, contact vs. both).
		n = 104 cells, SCs	Friedman test	Friedman test: p>0.999 (ns)
		n = 112 cells, SCm	Friedman test	Friedman test: p=0.4063 (ns)
		n = 120 cells, dPAG	Friedman test	Friedman test: p=0.0079 (**). Dunn's multiple comparisons, p=0.0058 (**, stim vs. contact), p=0.5257 (ns, stim vs. both), p=0.2441 (ns, contact vs. both).
		n = 330 cells, vPAG	Friedman test	Friedman test: p=0.1893 (ns)
		n = 437 cells, MRN	Friedman test	Friedman test: p=0.1074 (ns)
		n = 130 cells, SN	Friedman test	Friedman test: p=0.0171 (*). Dunn's multiple comparisons, p=0.0130 (*, stim vs. contact), p=0.4613 (ns, stim vs. both), p=0.4613 (ns, contact vs. both).
		n = 164 cells, RN	Friedman test	Friedman test: p=0.0006 (***). Dunn's multiple comparisons, p=0.0004(***, stim vs. contact), p=0.2053 (ns, stim vs. both), p=0.1405 (ns, contact vs. both).
		n = 207 cells, RRF	Friedman test	Friedman test: p<0.0001 (****). Dunn's multiple comparisons, p<0.0001 (****, stim vs. contact), p=0.0002 (***, stim vs. both), p<0.0001 (****, contact vs. both).
2	b	n = 159 cells, 9 mice; 10 trials per condition	Two-way RM ANOVA with Sidak's multiple comparisons test	Interaction Factor: F(158, 3021)= 14.33, p<0.0001 (***), Time factor: F(158, 3021)=18.58, p<0.0001 (***), Column Factor: F(1, 3021)=5.238, p=0.0222 (*). Sidak's multiple comparisons test (compare across cells, pie chart insert percent active)
	c	n = 159 cells, 9 mice; 10 trials per condition	Two-way RM ANOVA with Sidak's multiple comparisons test	Interaction Factor: F(158, 3021)= 97.69, p<0.0001 (***), Time factor: F(158, 3021)=160.2, p<0.0001 (***), Column Factor: F(1, 3021)=5782, p<0.0001 (***). Sidak's multiple comparisons test (compare across cells, pie chart insert percent active)
	d	n = 159 cells, 9 mice; 10 trials per condition	Two-way RM ANOVA with Sidak's multiple comparisons test	
	e	n = 52 cells, 4 mice; 10 trials per condition	Two-way RM ANOVA	Interaction Factor: F(51, 988)= 16.06, p<0.0001 (***), Time factor: F(51, 988)=17.77, p<0.0001 (***), Column Factor: F(1, 988)=5205, p<0.0001 (***).
			Two-way RM ANOVA	Interaction Factor: F(51, 988)= 11.10, p<0.0001 (***), Time factor: F(51, 988)=25.84, p<0.0001 (***), Column Factor: F(1, 988)=895.3, p<0.0001 (***).
	j	n=7 Vgat, n=7 Vglut2 mice	Two-tailed Mann-Whitney test	p=0.0006 (***).
	l	n = 47 cells, 9 mice; 10 trials per condition	Two-way RM ANOVA	
	m	n=40 interneuron, n=73 multimodal cells from 4 mice	Two-tailed Mann-Whitney test	p=0.0008 (***).
3	b	n = 142 cells, 7 mice; 10 trials per condition	Two-way RM ANOVA with Sidak's multiple comparisons test	Interaction Factor: F(141, 5396)= 121.4, p<0.0001 (***), Time factor: F(141, 5396)=149.1, p<0.0001 (***), Column Factor: F(1, 5396)=12805, p<0.0001 (***).
	l	n = 62 cells, 4 mice; 10 trials per condition	Two-way RM ANOVA	Interaction Factor: F(60, 2318)= 82.59, p<0.0001 (***), Time factor: F(60,
4	c	n=5 mate, n=5 control mice	Two-tailed unpaired t-test	p=0.0003 (***)
	k	n = 5 mice	Two-tailed Wilcoxon matched-pairs signed rank test	P=0.0625
5	b	n=7 mice	Two-tailed Mann Whitney test	p=0.5350
	c	n=7 mice	Two-tailed Mann Whitney test	p>0.9999
	d	n=7 mice	Two-tailed Mann Whitney test	p=0.0006 (***).
	e	n=7 mice	Two-tailed Mann Whitney test	p=0.0006 (***).
	f	n = 5 mice	Two-tailed paired t-test	p=0.009 (**).
	g	n = 5 mice	Friedman test	Friedman test: Reach p=0.0035 (**). Dunn's multiple comparisons, p>0.9999 (ns, sham vs. 1), p>0.9999 (ns, sham vs. 5), p=0.2781 (ns, sham vs. 10), p=0.0137 (*, sham vs. 20), p>0.9999 (ns, 1 vs. 5), p=0.2781 (ns, 1 vs. 10), p=0.0137 (*, 1 vs. 20), p>0.9999 (ns, 5 vs. 10), p>0.9999 (ns, 5 vs. 20), p>0.9999 (ns, 10 vs. 20)
		n = 5 mice	Friedman test	Friedman test: Mount p=0.0013 (**). Dunn's multiple comparisons, p>0.9999 (ns, sham vs. 1), p>0.9999 (ns, sham vs. 5), p=0.2145 (ns, sham vs. 10), p=0.0373 (*, sham vs. 20), p>0.9999 (ns, 1 vs. 5), p=0.0373 (*, 1 vs. 10), p=0.0047 (**, 1 vs. 20), p>0.9999 (ns, 5 vs. 10), p=0.4550 (ns, 5 vs. 20), p>0.9999 (ns, 10 vs. 20)
	i	n=6 mice	One-way repeated measure ANOVA with Tukey's multiple comparisons test	One way-ANOVA test: F(1.243, 6.217)=16.94, p=0.0047 (**). Tukey's multiple comparisons test: p=0.9977 (ns, pre vs. app), p=0.0317 (*,pre vs. contact), p=0.0246 (*, pre vs. sniff), p=0.0432 (*, pre vs. mount), p=0.0430 (*, app vs. contact), p=0.0287 (*, app vs. sniff), p=0.0497 (*, app vs. mount), p=0.6409 (ns, contact vs. sniff), p=0.6589 (ns, contact vs. mount), p=0.4284 (ns, sniff vs. mount
	k	n=6 mice	One-way repeated measure ANOVA with Tukey's multiple comparisons test	One way-ANOVA test: F(1.657, 8.287)= 24.27, p=0.0005 (***). Tukey's multiple comparisons test: p=0.0032 (**,wall vs. mount), p=0.0021 (**,groom vs. mount), p=0.0071 (*, allogroom vs. mount).

	l	n=6 mice	Two-tailed paired t-test	p=0.0020 (**).
	m	n=6 mice	One-way repeated measure ANOVA with Tukey's multiple comparisons test	One way-ANOVA test: F(1.257, 6.283)=32.85, p=0.0008 (***). Tukey's multiple comparisons test: p=0.0473 (*,pre vs. ball), p=0.0077 (**,pre vs. pc), p=0.0033 (*, pre vs. female), p=0.7232 (ns, ball vs. pc), p=0.0038 (**, ball vs. female), p=0.0224 (*, pc vs. female)
	n	n=5 mice (1 did not ejaculate)	Two-tailed paired t-test	p=0.0027 (**).
EDF 1	d	n=9 mice	Two-tailed paired t-test	p=0.0133 (*)
	f	n=5 mice	Two-tailed paired t-test	p=0.0007 (***)
EDF 3	c	n=2300 cells; 14 mice; 10 trials per condition	Friedman test with Dunn's multiple comparisons test	Friedman test: p<0.0001 (***). Dunn's multiple comparisons test: Cluster a: p>0.9999 (ns, stim vs. both), p<0.0001 (***, contact vs. both).Cluster b: p>0.9999 (ns, stim vs. both), p<0.0001 (***, contact vs. both). Cluster c: p<0.001 (***, stim vs. both), p=0.0213 (*, contact vs. both). Cluster d: p<0.001 (***, stim vs. both), p>0.9999 (ns, contact vs. both). Cluster e: p<0.001 (***, stim vs. both), p<0.001 (***, contact vs. both). Cluster f: p<0.001 (***, stim vs. both), p<0.001 (***, contact vs. both).
EDF 4	c	n= 49 cells, 3 mice; 10 trials per condition		ns
	d	n= 49 cells, 3 mice; 10 trials per condition		ns
	e	n= 35 cells, 3 mice; motion	Two-tailed Wilcoxon matched-pairs signed rank test	p=0.0003 (***)
		n= 98 cells, 4 mice; temperature	Friedman test with Dunn's multiple comparisons test	p=0.3755 (ns)
		n= 80 cells, 3 mice; texture	Friedman test with Dunn's multiple comparisons test	p=0.3604 (ns)
		n= 80 cells, 3 mice; pressure	Friedman test with Dunn's multiple comparisons test	p=0.5999 (ns)
	g	n = 71 cells, 3 mice	Friedman test with Dunn's multiple comparisons test	Friedman test: p<0.0001 (****). Dunn's multiple comparisons, p=0.0217 (*, w/ vs. w/o), p<0.0001 (****, w/ vs. numb), p=0.0448 (*, w/o vs. numb)
	h	n = 71 cells, 3 mice	Friedman test with Dunn's multiple comparisons test	Friedman test: p<0.0001 (****). Dunn's multiple comparisons, p<0.0001 (****, 1 vs. multi), p<0.0001 (****, 1 vs. face), p=0.0109 (*, multi vs. face)
	m	n = 94 cells	Two-tailed Wilcoxon matched-pairs signed [illegible]	p =<0.0001
EDF 6	b	n=6 mice	Two-tailed unpaired t-test	p = 0.0114 (*)
	d	n=6 mice	Two-tailed Wilcoxon matched-pairs signed rank test	p=0.0625
	e	n=6 mice	Two-tailed Wilcoxon matched-pairs signed rank test	p=0.4375
	f	n=6 mice	Two-tailed Wilcoxon matched-pairs signed rank test	p=0.0312 (*)
	g	n=6 mice	Two-tailed Wilcoxon matched-pairs signed rank test	p=0.1250 (ns)
	h	n=6 mice	Two-tailed Wilcoxon matched-pairs signed rank test	p=0.0417 (*)
	k	n=6 mice	Two-tailed Wilcoxon matched-pairs signed rank test	p=0.4375
	l	n=6 mice	Two-tailed Wilcoxon matched-pairs signed rank test	p=0.2188
	m	n=6 mice	Two-tailed Wilcoxon matched-pairs signed rank test	p=0.0312 (*)
	n	n=6 mice	Two-tailed Wilcoxon matched-pairs signed rank test	p=0.0625
	o	n=6 mice	Two-tailed Wilcoxon matched-pairs signed rank test	p>0.9999
	q	n = 3 mice	Two-way RM ANOVA with Sidak's multiple comparisons test	Interaction Factor: F(2, 154)= 0.814, p=0.4446 (ns), Condition Factor (stim v. sham): F(1, 154)=19.72, p<0.0001 (***), Trials Factor: F(2, 154)=1.488, p=0.2291(ns). Sidak's multiple comparisons test.
EDF 7	b	n=5 mice	Two-tailed paired t-test	p=0.0056 (**, stim vs. sham, reach)
		n=5 mice	Two-tailed paired t-test	p=0.0193 (*, stim vs. sham, mount)
	c	n=5 mice	Two-tailed paired t-test	p=0.0026 (**, stim vs. sham, reach)
		n=5 mice	Two-tailed Wilcoxon matched-pairs signed rank test	p=0.0625 (ns, stim vs. sham, mount)
	d	n=5 mice	Two-tailed paired t-test	p=0.0126 (*, stim vs. sham, reach)
		n=5 mice	Two-tailed paired t-test	p=0.0040 (**, stim vs. sham, mount)
	e	n=5 mice	Two-tailed Wilcoxon matched-pairs signed rank test	p=0.75 (ns, stim vs. sham, reach)
		n=5 mice	Two-tailed paired t-test	p=0.0790 (ns, stim vs. sham, mount)
	q	n=5 mice	Two-tailed paired t-test	p = 0.0075 (**)
	r	n=5 mice	Two-tailed paired t-test	p=0.0332 (*)

**All panels**	**Data were first tested for normality using a Shapiro-Wilk test and tested for homogeneity of variance using a Levene's test. If data met normality and homogeneity of variance assumptions, parametric tests were used (for example, Student's t test, one-way or repeated-measures ANOVA with Tukey's multiple comparison). If not, non-parametric tests were used (for example, Mann-Whitney U test or Kruskal–Wallis with Dunn's multiple comparison test). Paired tests were used to compare within-group repeated-measures data (for example, Wilcoxon signed rank test and Friedman test with Dunn's multiple comparison). All statistical tests were two-sided. Significance levels are indicated as follows: ∗p < 0.05; ∗∗ p < 0.01; ∗∗∗ p < 0.001. For all representative images, similar results were obtained in at least three independent experiments.**

Supplementary Table 2: Statistical Analyses
