# Supplementary material for: A circuit that integrates drive state and social contact to gate mating
Source: Nature. 2025 Sep 3;646(8084):394–403. doi: 10.1038/s41586-025-09327-x (PMC12507686; doi:10.1038/s41586-025-09327-x)
Supplement: Supplementary file 2 — Reporting Summary [file 41586_2025_9327_MOESM2_ESM.pdf]

nature portfolio

Corresponding author(s): David Anderson

Last updated by author(s): Jun 2, 2025

Reporting Summary

Nature Portfolio wishes to improve the reproducibility of the work that we publish. This form provides structure for consistency and transparency in reporting. For further information on Nature Portfolio policies, see our Editorial Policies and the Editorial Policy Checklist.

Statistics

For all statistical analyses, confirm that the following items are present in the figure legend, table legend, main text, or Methods section.

n/a	Confirmed	
☐	☒	The exact sample size (*n*) for each experimental group/condition, given as a discrete number and unit of measurement
☐	☒	A statement on whether measurements were taken from distinct samples or whether the same sample was measured repeatedly
☐	☒	The statistical test(s) used AND whether they are one- or two-sided<br>*Only common tests should be described solely by name; describe more complex techniques in the Methods section.*
☐	☒	A description of all covariates tested
☐	☒	A description of any assumptions or corrections, such as tests of normality and adjustment for multiple comparisons
☐	☒	A full description of the statistical parameters including central tendency (e.g. means) or other basic estimates (e.g. regression coefficient) AND variation (e.g. standard deviation) or associated estimates of uncertainty (e.g. confidence intervals)
☐	☒	For null hypothesis testing, the test statistic (e.g. *F*, *t*, *r*) with confidence intervals, effect sizes, degrees of freedom and *P* value noted<br>*Give P values as exact values whenever suitable.*
☒	☐	For Bayesian analysis, information on the choice of priors and Markov chain Monte Carlo settings
☒	☐	For hierarchical and complex designs, identification of the appropriate level for tests and full reporting of outcomes
☒	☐	Estimates of effect sizes (e.g. Cohen's *d*, Pearson's *r*), indicating how they were calculated

Our web collection on statistics for biologists contains articles on many of the points above.

Software and code

Policy information about availability of computer code

Data collection	OpenEphys for electrophysiology. Streampix 7 and python for video acquisition. Avisoft-RECORDER USGH for audio recording. Synapse for fiber photometry recording.
Data analysis	MATLAB 2020a, Python 3.8 and GraphPad Prism 9 for data and statistical analysis. Kilosort 2.5 for spike sorting and Phy v2 for manual curation. DeepSqueak v3 for USV detection. Facemap for behavioral analysis. MARS and Bento for behavior annotations. ImageJ, SHARCQ and AllenCCF for image analysis.

For manuscripts utilizing custom algorithms or software that are central to the research but not yet described in published literature, software must be made available to editors and reviewers. We strongly encourage code deposition in a community repository (e.g. GitHub). See the Nature Portfolio guidelines for submitting code & software for further information.

Data

Policy information about availability of data

All manuscripts must include a data availability statement. This statement should provide the following information, where applicable:

- Accession codes, unique identifiers, or web links for publicly available datasets
- A description of any restrictions on data availability
- For clinical datasets or third party data, please ensure that the statement adheres to our policy

The data that support the findings of this study are available from the corresponding author upon request.

Research involving human participants, their data, or biological material

Policy information about studies with human participants or human data. See also policy information about sex, gender (identity/presentation), and sexual orientation and race, ethnicity and racism.

Reporting on sex and gender	N/A
Reporting on race, ethnicity, or other socially relevant groupings	N/A
Population characteristics	N/A
Recruitment	N/A
Ethics oversight	N/A

Note that full information on the approval of the study protocol must also be provided in the manuscript.

Field-specific reporting

Please select the one below that is the best fit for your research. If you are not sure, read the appropriate sections before making your selection.

☒ Life sciences ☐ Behavioural & social sciences ☐ Ecological, evolutionary & environmental sciences

For a reference copy of the document with all sections, see nature.com/documents/nr-reporting-summary-flat.pdf

Life sciences study design

All studies must disclose on these points even when the disclosure is negative.

Sample size	No statistics were used to determine sample sizes. Sizes were determined based on prior experiments to sufficiently detect meaningful biological differences with good reproducibility (Karigo et al., 2020 and Yang et al., 2022) cited in the manuscript.
Data exclusions	Animals in which the virus injection and/or implantation missed the target brain region were excluded from analysis based on predetermined criterion including lack of expression or off-target labeling.
Replication	All experiments were repeated at least two times with separate cohorts of animals, and reproducibility was confirmed.
Randomization	For functional experiments, animals in a cage were randomly assigned to either control or experimental group. The order of presented conditions were randomized in head-fixed and freely moving experiments.
Blinding	The experimenter was blind to experimental or control group during data collection for behavioral testing and data analysis.

Reporting for specific materials, systems and methods

We require information from authors about some types of materials, experimental systems and methods used in many studies. Here, indicate whether each material, system or method listed is relevant to your study. If you are not sure if a list item applies to your research, read the appropriate section before selecting a response.

Materials & experimental systems

n/a	Involved in the study	
☐	☒	Antibodies
☒	☐	Eukaryotic cell lines
☒	☐	Palaeontology and archaeology
☐	☒	Animals and other organisms
☒	☐	Clinical data
☒	☐	Dual use research of concern
☒	☐	Plants

Methods

n/a	Involved in the study	
☒	☐	ChIP-seq
☒	☐	Flow cytometry
☒	☐	MRI-based neuroimaging

Antibodies

Antibodies used	anti-DsRed (Takara Bio, Cat# 632496, AB_10013483), anti-Fos (Santa Cruz, sc52-g, AB_2629503), Alexa Fluor 594 donkey anti-rabbit

Antibodies used	(Invitrogen, A-21207, AB_141637), and Alexa Fluor 647 donkey anti-goat (Invitrogen, A-21447,AB_141844).
Validation	Commercially available and validated in prior studies. Fos antibody (PMID: 24739975), DsRed antibody (PMID: 31810837).

Animals and other research organisms

Policy information about studies involving animals; ARRIVE guidelines recommended for reporting animal research, and Sex and Gender in Research

Laboratory animals	Study used male and female mice, aged 8 to 24 weeks. The following mouse lines were used: Esr1flp knock-in (JAX no. 036028), Slc32a1(Vgat)cre knock-in (Jax no. 028862), Slc17a6(Vglut2)cre knock-in (Jax no. 028863), Ai6 (Jax no. 007906), C57BL/6N (Jax no. 005304) , BALB/c (Jax no. 000651), and Pth2cre knock-in mice (GemPharmatechm, no. T054004). Mice were housed in ventilated micro-isolator cages in a temperature and humidity- controlled environment under a reverse 12-h light/dark cycle. Food and water was provided ad libitum. All mice used in this study, including wild-type and transgenic mice, were bred at Caltech. Mouse cages were changed weekly.
Wild animals	No wild animals were used in this study.
Reporting on sex	Only male mice were used for neural recording experiments in this study.
Field-collected samples	No field collected samples were used in the study.
Ethics oversight	All experimental procedures involving the use of live animals or their tissues were performed in accordance with the NIH guidelines and approved by the Institutional Animal Care and Use Committee (IACUC) and the Institutional Biosafety Committee at the California Institute of Technology.

Note that full information on the approval of the study protocol must also be provided in the manuscript.

Plants

Seed stocks	N/A
Novel plant genotypes	N/A
Authentication	N/A
